# LiCl attenuates impaired learning and memory of APP/PS1 mice, which in mechanism involves α7 nAChRs and Wnt/β‐catenin pathway

**DOI:** 10.1111/jcmm.17006

**Published:** 2021-10-28

**Authors:** Jie Xiang, Long‐Yan Ran, Xiao‐Xiao Zeng, Wen‐Wen He, Yi Xu, Kun Cao, Yang‐Ting Dong, Xiao‐Lan Qi, Wen‐Feng Yu, Yan Xiao, Zhi‐Zhong Guan

**Affiliations:** ^1^ Department of Pathology Guizhou Medical University and the Affiliated Hospital of Guizhou Medical University Guiyang P.R. China; ^2^ Key Laboratory of Endemic and Ethnic Diseases Ministry of Education Guizhou Medical University Guiyang P.R. China; ^3^ Provincial Key Laboratory of Medical Molecular Biology Guiyang P.R. China

**Keywords:** APP/PS1 mice, AβOs, LiCl, Wnt/β‐catenin signalling pathway, α7 nAChR

## Abstract

We examined the mechanism by which lithium chloride (LiCl) attenuates the impaired learning capability and memory function of dual‐transgenic APP/PS1 mice. Six‐ or 12‐month‐old APP/PS1 and wild‐type (WT) mice were randomized into four groups, namely WT, WT+Li (100 mg LiCl/kg body weight, gavage once daily), APP/PS1 and APP/PS1+Li. Primary rat hippocampal neurons were exposed to β‐amyloid peptide oligomers (AβOs), LiCl and/or XAV939 (inhibitor of Wnt/β‐catenin) or transfected with small interfering RNA against the β‐catenin gene. In the cerebral zone of APP/PS1 mice, the level of Aβ was increased and those of α7 nicotinic acetylcholine receptors (nAChR), phosphor‐GSK3β (ser9), β‐catenin and cyclin D1 (protein and/or mRNA levels) reduced. Two‐month treatment with LiCl at ages of 4 or 10 months weakened all of these effects. Similar expression variations were observed for these proteins in primary neurons exposed to AβOs, and these effects were attenuated by LiCl and aggravated by XAV939. Inhibition of β‐catenin expression lowered the level of α7 nAChR protein in these cells. LiCl attenuates the impaired learning capability and memory function of APP/PS1 mice via a mechanism that might involve elevation of the level of α7 nAChR as a result of altered Wnt/β‐catenin signalling.

## 1 INTRODUCTION

As the most frequently occurring ageing‐related dementia, Alzheimer's disease (AD) features progressive loss of cognitive abilities, in particular learning and memory.^1^ The hallmarks of this disease in the brain include intracellular tau (highly phosphorylated)‐containing neurofibrillary tangles, extracellular β‐amyloid peptide (Aβ)‐containing amyloid plaques, an excessive loss of synapses and deficient cholinergic transmission.^2^

The ‘cholinergic hypothesis’ proposes that among AD patients observed, decreased cholinergic innervation is a contributing factor of cognitive degradation.^3^ The nicotinic acetylcholine receptors (nAChRs) of neurons participate in cognitive processing, as well as learning and memory. Expression alterations in these receptors are associated with the impaired cholinergic neurotransmission^4^ and may be responsible for complex neuronal diseases such as AD and Parkinson disease.^5^

Indeed, nAChRs level is lowered in the cerebral zone of the AD patients and of animal models of this disease, as well as in isolated neuronal cells exposed to Aβ.^6^, ^7^ α7, the main subtype of nAChR present in both neuronal and non‐neuronal cells of the human brain consists mainly of five α7 subunits and is considered to be one of the receptors most closely associated with the AD contraction.^8^ Aβ_1‐40_ and Aβ_1‐42_ bind to α7 nAChR with an affinity in the nanomolar range, resulting in gradual depletion and inactivation of this receptor.^9^

In addition to the toxic consequences due to the direct nAChRs binding by Aβ, other subcellular events have been associated with AD, including down‐regulation of canonical axis Wnt/β‐catenin, which might influence the stability of these receptors. When Wnt receptors are not activated, the β‐catenin bound by Axin is phosphorylated in succession via glycogen synthase kinase‐3β (GSK3β) and casein kinase 1 at a range of Ser/Thr residues (regularly spaced), which are located at the N‐terminus of the β‐catenin. Consequently, ubiquitination and targeting of β‐catenin are initiated for fast disruption by proteasome, thereby depressing the expression of downstream targets such as cyclin D1.^10^

Interestingly, Wnt/β‐catenin signalling is closely linked to learning capability and memory function among mammals and this signalling is impaired both in the brains of AD patients,^11^ as well as animal AD models.^12^ This dysfunction exerts a crucial effect on the neuronal degeneration, as well as the impairment of synapses, creating a deleterious pathway that leads to dementia.^13^ Notably, nAChRs are present both on presynaptic^14^ and on postsynaptic^15^ membranes, and loss of these receptors leads inevitably to abnormalities in synapses. Furthermore, when the Wnt signalling pathway is activated persistently via ligand medication or suppressor deactivation, it overcomes the toxic effects of Aβ and improves cognitive performance in patients with AD.^16^, ^17^

As is widely acknowledged, lithium is the preferred mood‐stabilizing agent for maintaining bipolar disorder.^18^ Apart from stabilizing moods, lithium can also resist suicidal thoughts, regulate immunity and protect nerve system,^19^ which, during the last decade, has come to be regarded as a neuroprotective agent and is now widely used in studies of neurodegenerative diseases, including AD.^20^ It is noteworthy in this context that the incidence of AD among patients with bipolar disorder who have been taking lithium is lower than among those not undergoing lithium therapy.^18^ Lithium chloride (LiCl) inhibits GSK3β, thereby reducing this activity in the brain of animal models of AD, while enhancing Wnt/β‐catenin signalling.^21^, ^22^

Accordingly, lithium may be of value in treating AD. However, it is currently unknown whether lithium can reverse the learning and memory impairments in murine AD model and, if so, whether this effect involves Wnt/β‐catenin signalling modulation, as well as consequent regulation of α7 nAChR. Here, we investigated these questions by treating both APP/PS1 transgenic mice and β‐amyloid peptide oligomer (AβO)‐exposed primary neurons using LiCl or XAV939, a small molecule selective inhibitor that promotes β‐catenin degradation and inhibits the transcription by β‐catenin,^23^ and then analysing the cognitive ability of these mice, as well as potential changes in Wnt/β‐catenin and α7 nAChR in both of these systems.

## 2 MATERIALS AND METHODS

### 2.1 Materials

LiCl, Aβ_1‐42_ and XAV939 (Sigma‐Aldrich Inc.); anti‐Aβ mouse monoclonal antibody (BioLegend Inc.); anti‐GSK3β, anti‐phosphor‐GSK3β (ser9), anti‐cyclin D1 and anti‐β‐catenin rabbit monoclonal antibodies, as well as horseradish peroxidase (HRP)‐conjugated anti‐rabbit IgG (Cell Signaling Technology); anti‐GAPDH rabbit polyclonal antibody (Genetex); anti‐nAChR α7 rabbit polyclonal antibody (Abcam); cell counting Kit‐8 (Dojindo Molecular Technologies); anti‐NeuN mouse monoclonal antibody (Merck Millipore); anti‐glial fibrillary acidic 113 protein (GFAP) rabbit monoclonal antibody (Dako); CY‐3‐labelled anti‐mouse IgG and 488‐labelled anti‐rabbit IgG (Thermo scientific Inc.); Aβ_42_ assaying kits (Thermo scientific Inc.); Lipofectamine RNAiMAX Reagent (Invitrogen Inc.); universal 2×PCR mastermix (TaqMan; Applied Biosystems); and the remaining chemicals were all procured from Sigma‐Aldrich.

### 2.2 Experimental animals

B6.Cg‐Tg (PSEN1dE9 and APP_SWE_) mice aged 4 months having a 85Dbo/Mmjax background were procured from Nanfang BioTechnology Co., Ltd. Meanwhile, wild‐type (WT) mice of identical strain were also procured from the same company. All mice were 20–30 g in body weight. Each male mouse was allowed to mate with four females. When the resulting pups were 12–20 days old, the tips of their tails were cut off for extraction of DNA and the polymerase chain reaction (PCR)‐based genotyping, where agarose gel electrophoresis (1.5%) was used for product analysis. For PCR primer design of target transcripts shown in Table 1, the entire cDNA sequences from GenBank were consulted.

**TABLE 1:** The primers used for amplification of mouse APP and PS1 cDNA

Gene	Sequence of primers	Length (bp)
APP	For 5′‐GACTGACCACTCGACCAGGTTCTG‐3′	400
	Rev 5′‐CTTGTAAGTTGGATTCTCATATCCG‐3′	
PS1	For 5′‐AATAGAGAACGGCAGGAGCA‐3′	608
	Rev 5′‐GCCATGAGGGCACTAATCAT‐3′	

The described experimental procedures in this study have all been approved by the Ethical Committee of Guizhou Medical University in China (no. 1702110).

Based on literature and the results of previous studies,^24^, ^25^ the 4‐ or 10‐month‐old WT and transgenic (APP/PS1) mice were randomized into four groups (six animals in each) for subsequent treatment for 2 months as follows: (1) the WT group: WT animals received gavage normal saline at about 0.4 ml once per day; (2) the WT+Li group: 100 mg/kg LiCl was administered to the WT animals; (3) the APP/PS1 group: normal saline was given to the animals with APP/PS1 double mutations in identical manner to group (1); (4) the APP/PS1+Li group: transgenic animals were treated with LiCl in the same manner as group (2).

### 2.3 Culturing and treatment of primary hippocampal neurons

Primary neurons were obtained from the cerebral zone of newborn Sprague‐Dawley rats employing minor modifications of a published procedure.^26^ For purity assessment of these primary neurons, immunofluorescent double staining was performed in succession using the anti‐NeuN mouse antibody, the CY‐3‐labelled anti‐mouse IgG (red), the anti‐GFAP rabbit antibody and a subsequent 488‐labelled anti‐rabbit IgG (green).

AβOs were prepared by a procedure described earlier^27^ and the suitable concentration for exposure chosen as also described previously.^26^ Initially, cell exposure to varying doses of LiCl (0–100 mmol/L) and XAV939 (0–50 μmol/L) was accomplished for different times. Thereafter, the cells were subjected to 2‐h incubation with CCK8 solution (10 μl), following which the absorption at 450 nm was determined.

Transfection with small interfering RNA (siRNA) was initiated by adding a mixture (1:1) of Lipofectamine RNAiMAX Reagent diluted in Opti‐MEM Medium (15 μl:250 μl) and the siRNA (For 5′‐GACUACCUGUUGUGGUUAAdTdT‐3′; Rev 5′‐UUAACCACAAAGGUAGUCdTdT‐3′), also diluted in Opti‐MEM Medium (1:50) for 5 min. Then, the cells were incubated with the siRNA‐Lipid complex for 48 h at 37°C.^28^

### 2.4 Morris water maze for spatial learning and memory

In a 25–26°C water‐filled round pool rendered opaque using milk powder, every mouse was compelled to find an underwater escape facility.^29^ In the familiarization and acquisition sessions, 60 s was offered for every mouse to seek the hidden facility, and then, 5 s was offered to keep seated on the facility. Afterwards, the mice returned to the original cage. In the retention session, where the facility was withdrawn from the pool, the path adopted by every mouse was video recorded for 60 s, in order to determine the time expenditure for swimming to the original facility location, and the number of passes across and the time expenditure at this location.

### 2.5 Determination of Aβ_42_ by ELISA

The murine cerebral level of Aβ_42_ was determined utilizing a murine Aβ_42_ ELISA kit as per the protocol of manufacturer.^29^ Briefly, after homogenization and centrifugation of the tissue, the pellets obtained were extracted with 5 M guanidine‐HCl. After dilution of aliquot protein from each extract to an ultimate volume (100 μl), 2‐h incubation was accomplished in plate wells under the room temperature (RT) condition. Next, the wells were rinsed, injected with solution of Detection Antibody (100 μl) and then incubated for one more hour at RT. Thereafter, the wells were again washed, followed by 30‐min incubation using solution of HRP‐linked antibody (100 μl) and four extra washes. The last step involved addition of stop solution and absorbance measurement (450 nm) with a spectrophotometer (Bio‐Rad Inc.).

### 2.6 Quantification of the levels of phosphor‐GSK3β (ser9), GSK3β, β‐catenin, cyclin D1 and α7 nAChR by Western blotting

Brain tissue or cultured cells in lysis buffer containing a mixture of protease inhibitors were disrupted in a glass homogenizer. The resulting homogenate was subjected to 20‐min centrifugation under 4°C and 15520 g conditions. For the resulting supernatants, the BCA protein assaying kit was utilized to measure the protein concentrations.^26^ Afterwards, protein isolation was performed via 10% SDS‐PAGE. Finally, a transfer unit (Bio‐Rad Inc.) was utilized to blot the protein isolates onto the polyvinylidene difluoride (PVDF) films.

To achieve relative protein quantization, the above PVDF membranes were then subjected to incubation using antibodies against phosphor‐GSK3β (ser9), GSK3β, β‐catenin, cyclin D1, α7 nAChR or GAPDH overnight at 4°C. Next, the membranes were rinsed and subjected to 60‐min incubation using secondary antibody conjugated to HRP. Subsequently, enhanced chemiluminescence kit (Millipore) was used to detect the protein bands, and visualization of the resulting signals was accomplished for 30 s–3 min by exposing to chemiluminescence film (hyperperformance). Signal intensity was quantified employing the Image J software.

### 2.7 Determination of the level of α7 nAChR mRNA by reverse transcription and quantitative real‐time PCR

Total RNA, which was extracted via Trizol Reagent from brain tissue or cells, was used to obtain cDNA by reverse transcription with the Prime Script™ RT Master Mix cDNA Synthesis Kit (Takara Bio.).^30^ A Sequence Detection System (ABI PRISM 7300; Applied Biosystems) was utilized to perform quantitative real‐time PCR as per the protocol of manufacturer. Then, the PCR analysis was accomplished with the aid of GeneAmp7300 SDS. The level of α7 nAChR transcripts was estimated using 2^−ΔΔCT^ (RQ value) and the formula ΔΔCT = ΔCT_target_−ΔCT_control_ = (CT_target_−CT_GAPDH_)−(CT_control_−CT_GAPDH_) with the error estimate being ΔΔCT plus and minus the standard deviation. The sequences of the PCR primers utilized are presented in Table 2. The level of GAPDH mRNA was used as the internal control.

**TABLE 2:** The primers used for amplification of mouse or rat α7 nAChR cDNA

Species	Gene	Sequence of primers	Length (bp)
Rat	α7 nAChR	For 5′‐CGGAGTGAAGAATGTTCGTTTT‐3′	241
		Rev 5′‐GAATATGCCTGGAGGGAGATAC‐3′	
Mouse	α7 nAChR	For 5′‐CACATTCCACACCAACGTCTT‐3′	106
		Rev 5′‐AAAAGGGAACCAGCGTACATC‐3′	
Rat	GAPDH	For 5′‐GACATGCCGCCTGGAGAAAC‐3′	92
		Rev 5′‐AGCCCAGGATGCCCTTTAGT‐3′	
Mouse	GAPDH	For 5′‐GGTTGTCTCCTGCGACTTCA‐3′	183
		Rev 5′‐TGGTCCAGGGTTTCTTACTCC‐3′	

### 2.8 Examination of senile plagues and α7 nAChR by immunohistochemical or immunofluorescent staining

Immunohistochemical staining for senile plagues and α7 nAChR in the brains of mice was accomplished by consulting prior literature.^31^ Initially, sections were deparaffinized and dehydrated. Next, they were subjected to 20‐min microwaving with pH 6.0 citric acid buffer (0.01 M) for antigen retrieval, followed by 30‐min placement in blocking medium (Dako Inc.) under RT condition. Afterwards, overnight incubation of sections was accomplished at 4°C using anti‐Aβ and anti‐α7 nAChR antibodies. The next day, incubation was further carried out using rabbit IgG or goat anti‐mouse biotinylated antibody under RT condition for 60 min. Afterwards, further incubation was accomplished using the avidin/biotinylated enzyme complex, followed by placement in diaminobenzidine‐containing peroxidase reaction buffer. Aided by Image Pro Plus, we quantified the number of senile plaques and integral optical density (IOD) of α7 nAChR staining in stochastically selected visual fields under a ×200 original magnification.

For immunostaining of α7 nAChR in isolated cells, seeding of primary neurons was done onto six‐well plates coated with polylysine, followed by three times of washing in PBS and paraformaldehyde fixation (4%). Then, these samples were subjected to 30‐min treatment with goat serum at RT and a subsequent overnight incubation with antibody against α7 nAChR at 4°C.^26^ The next day, incubation was further carried out with fluorescein isothiocyanate‐labelled anti‐rabbit goat IgG (488‐labelled) for 1 h at RT. Afterwards, the cells were subjected to three times of PBS rinsing and coverslipping with Vectashield (Vector Laboratories). Aided by Image Pro Plus, we quantified the IOD of immunofluorescent staining for α7 nAChR in stochastically selected visual fields under a ×400 original magnification.

### 2.9 Statistical analyses

For mice in various groups, the statistical values were represented as means ± SDs and compared via analysis of variance, and a subsequent post hoc (least significant difference) test. Bielschowsky procedure was employed to semi‐quantitate the Aβ plaques.^32^ The correlation of the level of α7 nAChR protein with spatial learning and memory was analysed employing the Pearson correlation test. SPSS 22.0 (SPSS Inc.) was used to carry out all analyses, and *p* values of <0.05 were regarded significantly different.

## 3 RESULTS

### 3.1 Confirmation of the APP/PS1 murine genotype

As shown in Figure S1, when the PCR amplification template was genomic DNA, the transgenic group exhibited products (sizes around 400 and 600 bp) that coincided separately with the APP and PS1 genes in size. However, this was not observed among the WT mice.

### 3.2 Spatial learning and memory in mice

**FIGURE 1:**
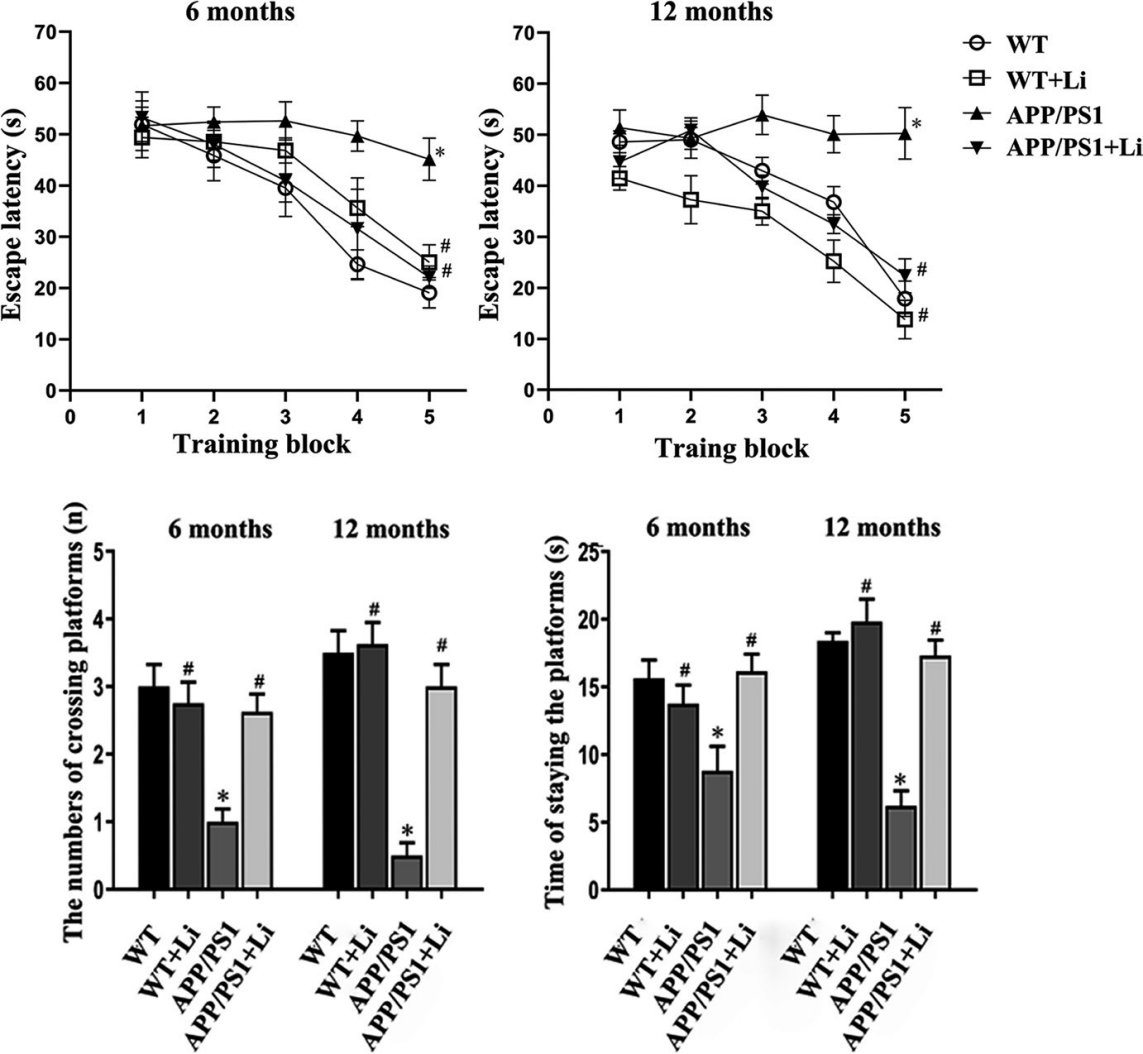
Morris water maze assessments of the learning capability and memory function among 6‐ or 12‐month‐old WT and dual‐transgenic APP/PS1 mice. The four groups were subjected to 2‐month gavage administration once daily as follows: WT: WT mice received physiological saline (PS); WT+Li: WT mice received LiCl (Li); APP/PS1: transgenic mice received PS; APP/PS1+Li: transgenic mice received Li. The values are represented as means ± SDs (*n* = 6), *p < 0.05 as compared to WT; ^#^ *p *< 0.05 as compared to APP/PS1 mice

According to the Morris water maze results displayed in Figure 1, both the number of times the original facility location was crossed and the time expenditure at this location were reduced, while the escape latency was increased in the 6‐ and 12‐month‐old APP/PS1 mice as compared to the corresponding WT mice. Treatment for the transgenic animals with LiCl for 2 months beginning at 4 or 10 months of age clearly improved both their learning capability and memory function.

### 3.3 Senile plaque count and level of Aβ_42_ in the cortex of mouse brains

**FIGURE 2:**
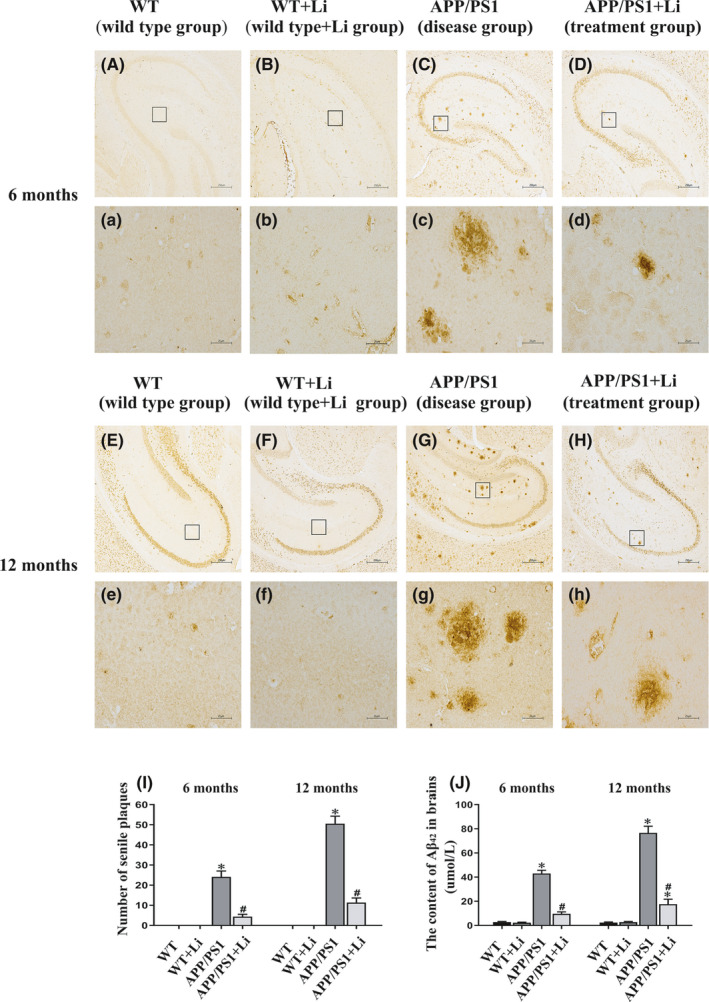
Senile plaques in the cerebral zone of 6‐ (A–D, a–d) and 12‐month‐old (E–H, e–h) WT and dual‐transgenic APP/PS1 mice. The four groups were subjected to 2‐month gavage administration once daily as follows: WT: WT mice received physiological saline (PS); WT+Li: WT mice received LiCl (Li); APP/PS1: APP/PS1 mice received PS; APP/PS1+Li: APP/PS1 mice received Li. A, a, E and e: the WT group; B, b, F and f: the WT+Li group; C, c, G and g: the APP/PS1 group; D, d, H and h: the APP/PS1+Li group. The quantitative data presented were obtained from six mice each group. Under light microscope with 40×, five non‐overlapping visual fields were randomly selected and counted from each section. Magnification: A–H, 40×, scale bar = 250 μm; a–h (magnification of the squares in A–H), 400×, scale bar = 25 μm. I: the number of senile plaques identified via immunohistochemistry. J: The content of Aβ_42_ in the brain as assayed by ELISA. The values are represented as means ± SDs (*n* = 6), *p < 0.05 as compared to WT; ^#^ *p *< 0.05 as compared to APP/PS1 mice

The brains of 6‐ (Figure 2A–D and a–d) or 12‐month‐old (Figure 2E–H and e–h) transgenic mice contained Aβ‐immunoreactive senile plaques, the number of which was markedly reduced by exposure to LiCl beginning at either 6 or 12 months of age. There were no detectable amyloid plaques in the cerebral zone of WT mice with or without exposure LiCl (Figure 2I).

The cortex of WT mice contained a very low level of Aβ_42_ that was unaffected by treatment with LiCl. In contrast, this level was much higher in untreated APP/PS1 animals, where the level was decreased even further by treatment with LiCl (Figure 2J).

### 3.4 Primary neuronal viability upon AβOs, LiCl and/or XAV939 exposures

As shown in Figure S2A–E, the immunostaining analysis of the primary neurons cultured using antibody, which were prepared from the cerebral hippocampal zone of neonatal rats, directed towards DAPI (a nucleus marker), GFAP (an astrocyte marker) and NeuN (a neuron marker), suggesting that these cells (about 90%) were neuronal. These primary neurons were assessed for viability following exposure to AβOs, LiCl and/or XAV939 utilizing the CCK‐8 test. Treatment with 10 mmol/L LiCl for 6 h or 1 μmol/L XAV939 for 24 h did not cause any significant cytotoxicity (Figure S2F,G). Exposure of these primary neurons to 0.5 μmol/L AβOs for 48 h (conditions chosen on the basis of an earlier study;^33^) reduced cell viability, a reduction that was attenuated by LiCl, but aggravated by XAV939 (Figure S2H).

### 3.5 Expression of proteins involved in Wnt signalling in the hippocampus and cortex of mouse brains and primary neurons

**FIGURE 3:**
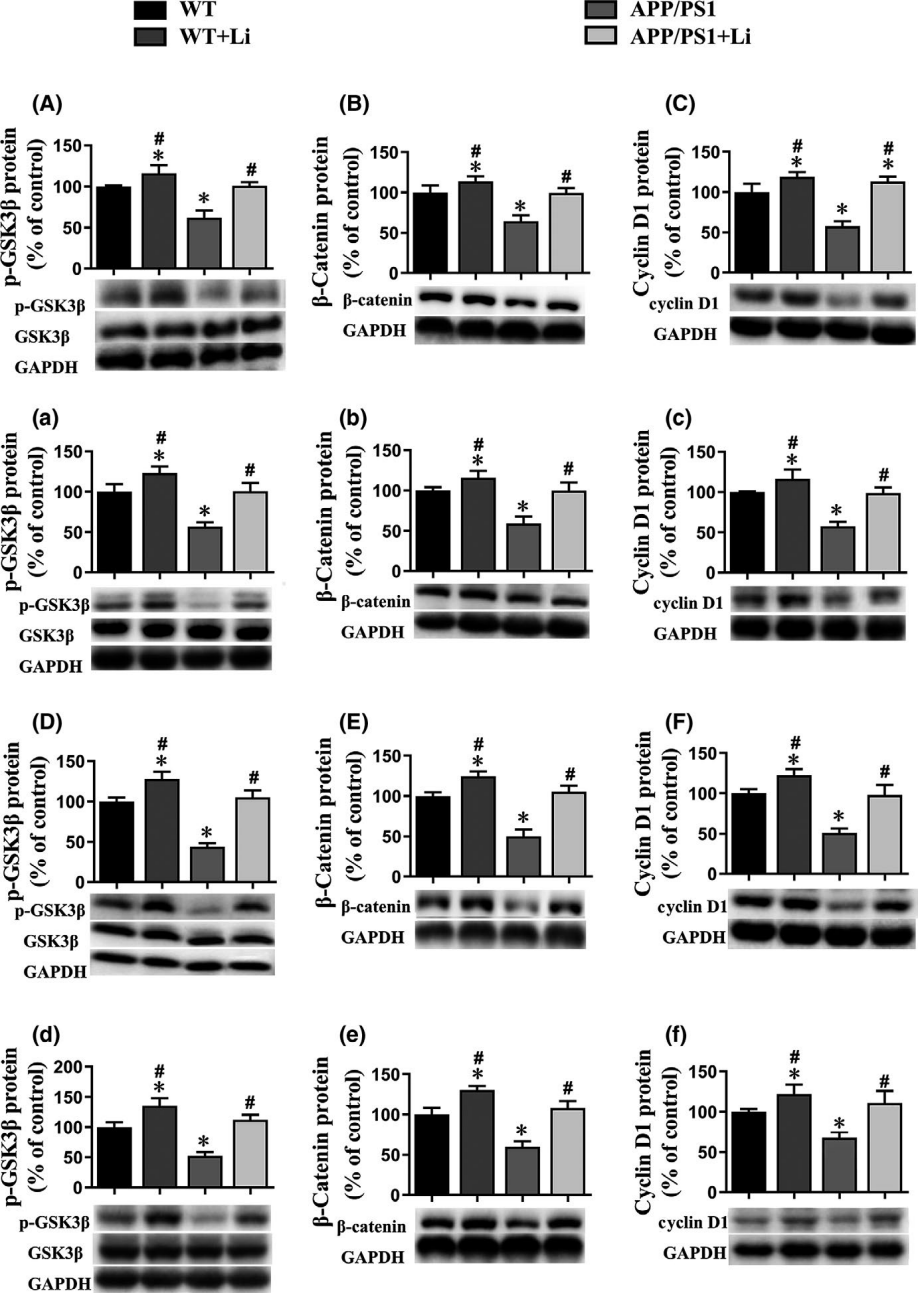
Expression of the p‐GSK3β, β‐catenin and cyclin D1 proteins in the hippocampus (A–F) and cortex (a–f) of the brains of 6‐ (A–C and a–c) or 12‐month‐old (D–F and d–f) WT and dual‐transgenic APP/PS1 mice. The four groups were subjected to 2‐month gavage administration once daily as follows: WT: WT mice received physiological saline (PS); WT+Li: WT mice received LiCl (Li); APP/PS1: APP/PS1 mice received PS; APP/PS1+Li: APP/PS1 mice received Li. The values are represented as means ± SDs (*n* = 6), *p < 0.05 as compared to WT; ^#^ *p *< 0.05 as compared to APP/PS1 mice

As Western blotting revealed, the phosphor‐GSK3β (ser9) (not total GSK3β), β‐catenin and cyclin D1 levels in the cerebral hippocampus (Figure 3A–C and D–F) or cortex (Figure 3a–c and d–f) of the APP/PS1 mice aged 6 (Figure 3A–C and a–c) or 12 (Figure 3D–F and d–f) months were all lower than the corresponding levels in the WT mice. Noteworthy is that such declines were mitigated by LiCl treatment for transgenic mice.

**FIGURE 4:**
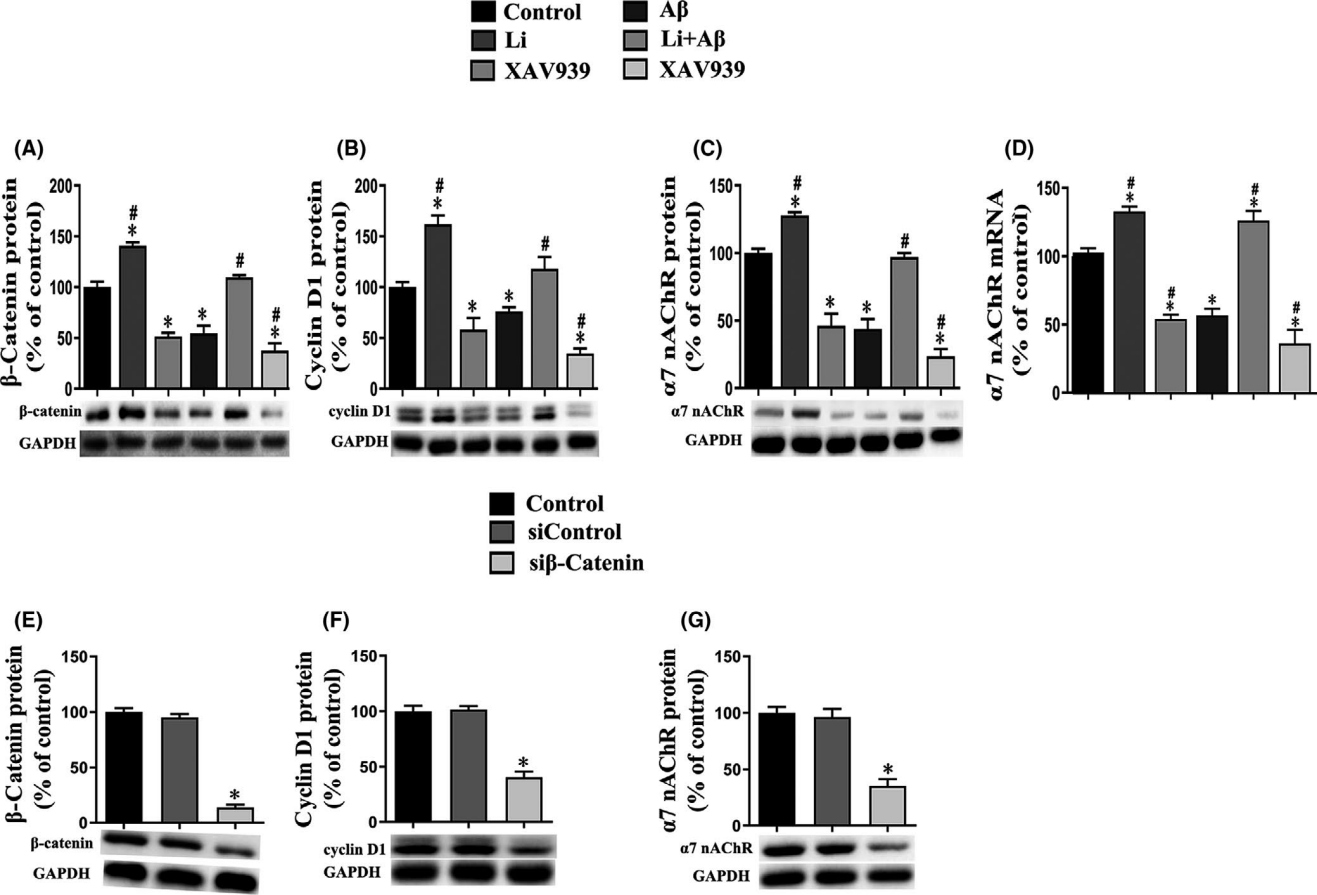
Expression of the β‐catenin (A and E) and cyclin D1 proteins (B and F) and α7 nAChR protein and mRNA (C, D and G) by primary hippocampus neurons. control: untreated primary neurons; Li: primary neurons exposed to LiCl (10 mmol/L) for 6 h; XAV939: primary neurons exposed to XAV939 (1 μmol/L) for 24 h; Aβ: primary neurons exposed to AβOs (0.5 μmol) for 48 h; Li+Aβ: primary neurons exposed to LiCl and AβOs; XAV939+Aβ: primary neurons exposed to XAV939 and AβOs; sicontrol: primary neurons transfected with sicontrol; siβ‐catenin: primary neurons transfected with small interfering RNA (siRNA) targeting the β‐catenin gene for 48 h. The values presented are the means ± SDs from three separate experiments. *p < 0.05 as compared to control group; ^#^ *p *< 0.05 as compared to the Aβ group

With regard to primary neurons, the levels of the β‐catenin and cyclin D1 proteins were enhanced by exposure to LiCl alone, whereas XAV939 reduced these levels. Exposure of primary neurons to AβOs alone also reduced these levels, an effect that was attenuated by LiCl and enhanced by XAV939 (Figure 4A,B).

Compared to the corresponding controls, transfection of primary neurons with siRNA targeting the β‐catenin gene reduced both the level of this protein and that of cyclin D1 (Figure 4E,F).

### 3.6 Expression of α7 nAChR mRNA and protein in the hippocampus and cortex of mouse brains and primary neurons

In comparison with the corresponding control group, the levels of both α7 nAChR protein and mRNA were enhanced by exposure of the neurons to LiCl alone and reduced by XAV939. Moreover, these levels were reduced by exposure to AβOs, a decline that was attenuated by LiCl, but enhanced by XAV939 (Figure 4C,D).

Moreover, transfection of neurons with siRNA targeting the β‐catenin gene reduced the level of this protein, as well as that of α7 nAChR (Figure 4G).

**FIGURE 5:**
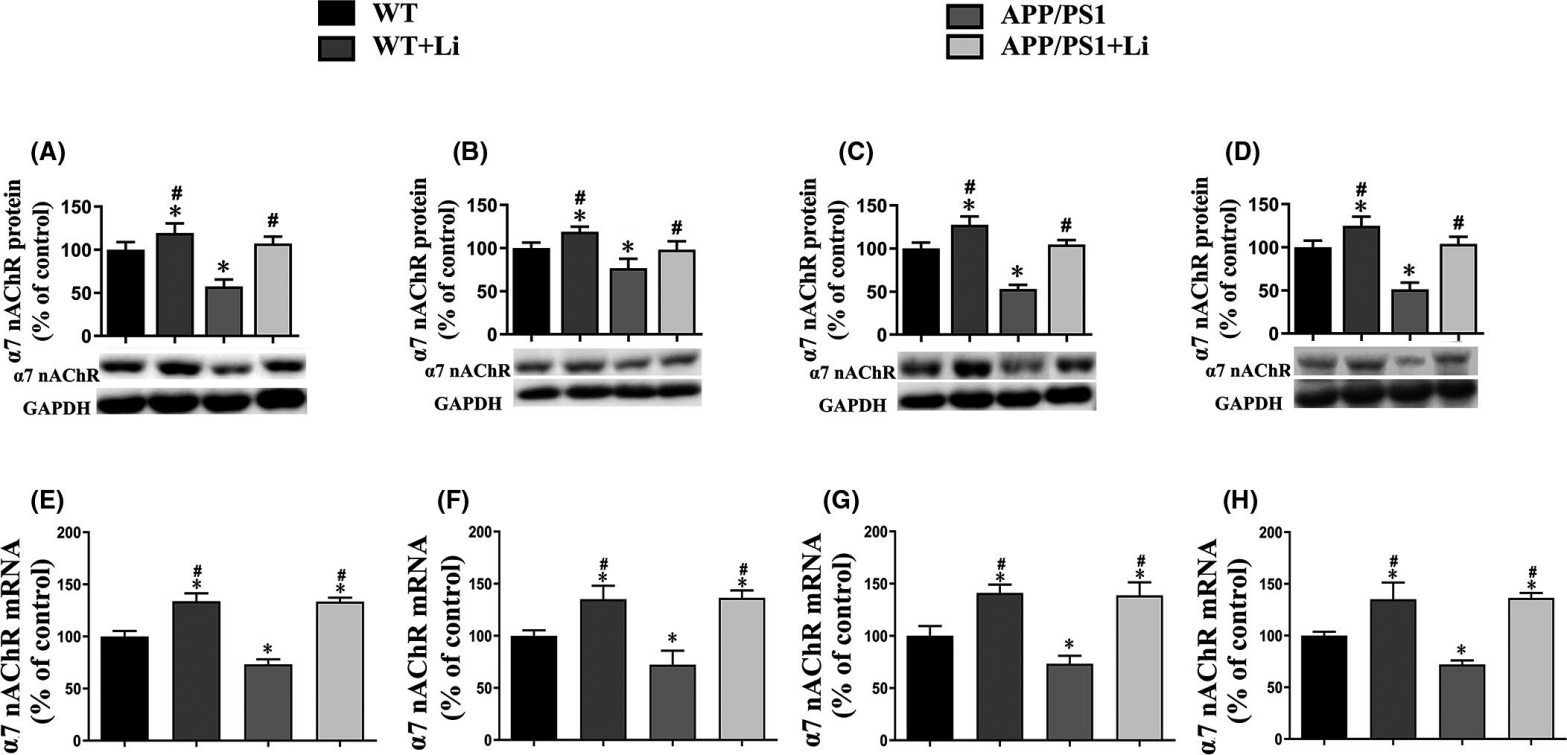
Protein and mRNA expressions of α7 nAChR in the hippocampus (A, C, E and G) and cortex (B, D, F and H) of the brains of 6‐ (A, B, E and F) and 12‐month‐old (C, D, G and H) WT and dual‐transgenic APP/PS1 mice. The four groups were subjected to 2‐month gavage administration once daily as follows: WT: WT mice received physiological saline (PS); WT+Li: WT mice received LiCl (Li); APP/PS1: APP/PS1 mice received PS; APP/PS1+Li: APP/PS1 mice received Li. The values are represented as means ± SDs (*n* = 6), *p < 0.05 as compared to WT; ^#^ *p *< 0.05 as compared to APP/PS1 mice

According to the results of real‐time PCR and Western blotting, declines in the protein (Figure 5A–D) and mRNA (Figure 5E–H) levels of α7 nAChR were noted in the hippocampus (Figure 5A,C,E,G) and cortex (Figure 5B,D,F,H) of APP/PS1 mice at 6 (Figure 5A,B,E,F) and 12 (Figure 5C,D,G,H) months of age. Administration of LiCl to either the WT or APP/PS1 animals elevated these levels in both of these areas of the brain.

**FIGURE 6:**
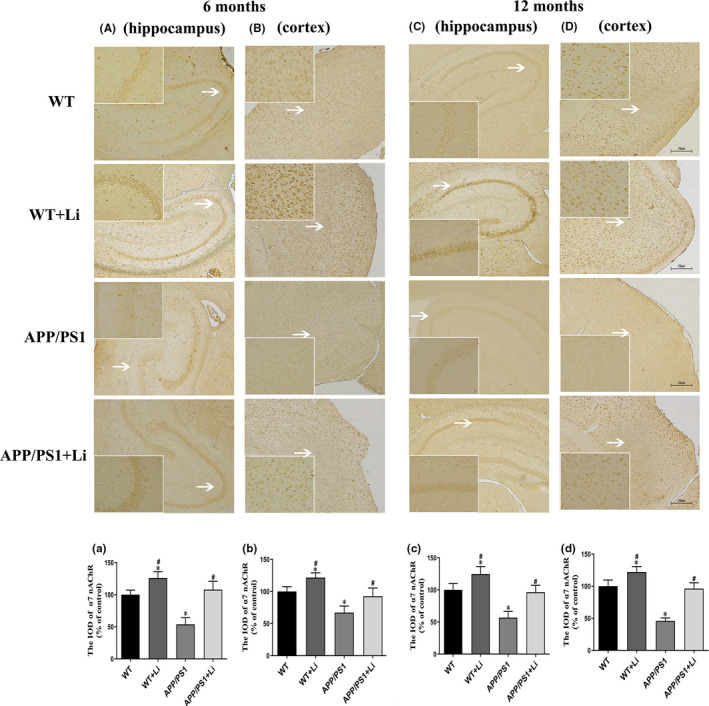
Immunohistochemical staining for α7 nAChR protein in the hippocampus (A and C) and cortex (B and D) of the brains of 6‐ (A and B) and 12‐month‐old (C and D) WT and dual‐transgenic APP/PS1 mice. The four groups were subjected to 2‐month gavage administration once daily as follows: WT: WT mice received physiological saline (PS); WT+Li: WT mice received LiCl (Li); APP/PS1: APP/PS1 mice received PS; APP/PS1+Li: APP/PS1 mice received Li. Magnification: A–D, 40×, scale bar = 250 μm or 200× (magnification of the white arrows in A–D). a–d: semiquantitative analysis of the integrated optical density (IOD) of α7 nAChR staining in the hippocampus (a, c) and cortex (b, d) of the 6‐ (a, b) and 12‐month‐old (c, d) mice in the different groups. The values are represented as means ± SDs (*n* = 6), *p < 0.05 as compared to WT; ^#^ *p *< 0.05 as compared to APP/PS1 mice

Semiquantitative immunohistochemical analysis of mouse brains revealed localization of α7 nAChR primarily at the plasma membrane and axon (Figure 6A–D and a–d). This immunostaining was less intense in the hippocampus (Figure 6A,a,C,c) and cortex (Figure 6B,b,D,d) of the APP/PS1 mice at 6 (Figure 6A,a,B,b) and 12 (Figure 6C,c,D,d) months of age than for the WT group. In both groups of animals, LiCl augmented the intensity of this staining.

**FIGURE 7:**
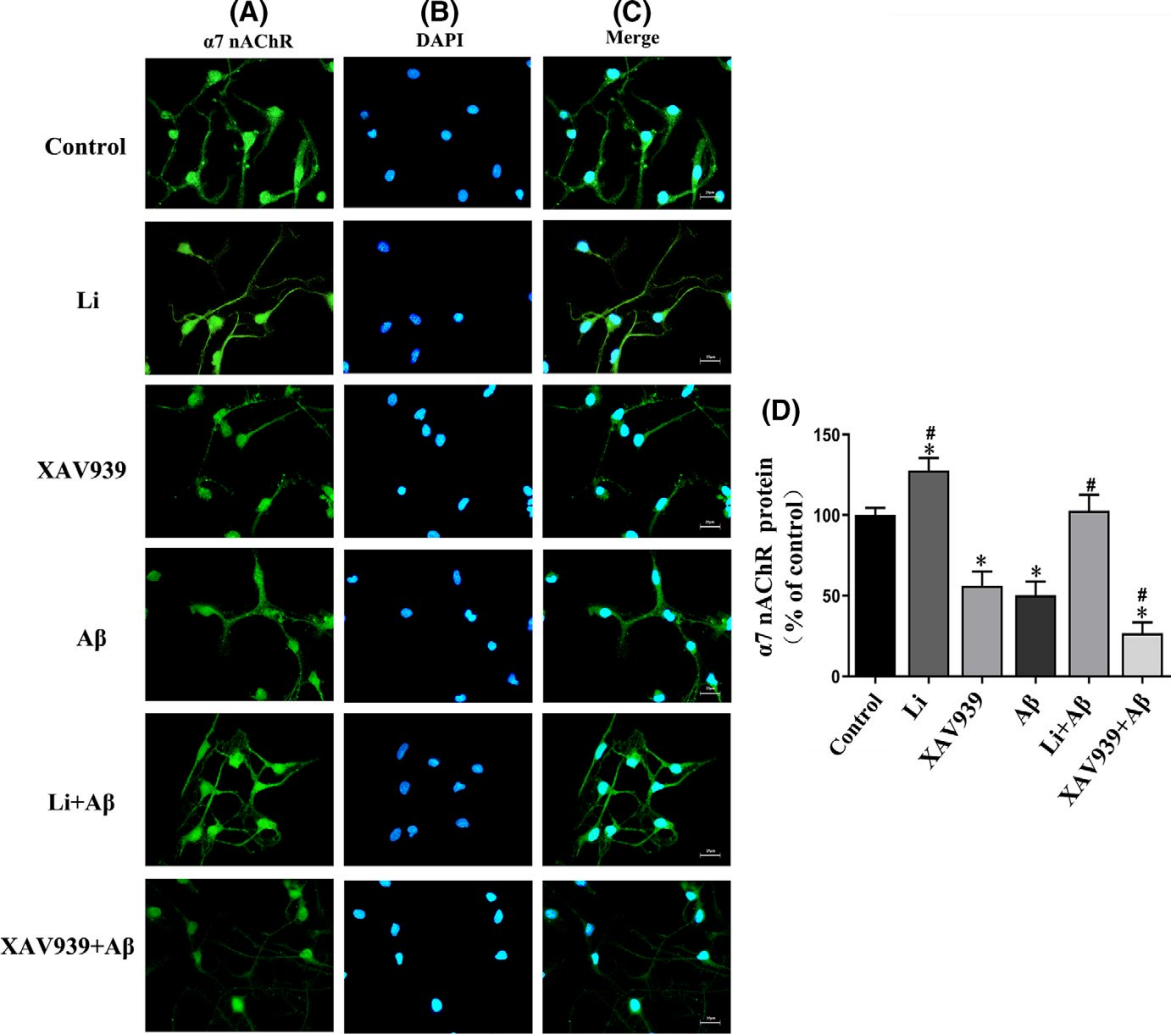
Immunofluorescent staining for α7 nAChR protein in primary hippocampus neurons. Control group: untreated primary neurons; Li: primary neurons exposed to LiCl (10 mmol/L) for 6 h; XAV939: primary neurons exposed to XAV939 (1 μmol/L) for 24 h; Aβ: primary neurons exposed to AβOs (0.5 μmol) for 48 h; Li+Aβ group: primary neurons exposed to LiCl and AβOs; XAV939+Aβ group: primary neurons exposed to XAV939 and AβOs. The neurons were stained using rabbit antibody against α7 nAChR (green), while staining of cellular nuclei was accomplished using DAPI (blue). D: semiquantitative analysis of the integrated optical density (IOD) for α7 nAChR staining. Magnification: 400×, scale bar = 25 μm. The values are represented as the means ± SDs from three separate experiments. *p < 0.05 as compared to the control group; ^#^ *p *< 0.05 as compared to the Aβ group

In the case of primary neurons, semiquantitative immunofluorescent analysis revealed localization of the α7 nAChR subunit mainly at the plasma membrane and axon (Figure 7A). Exposure to LiCl alone enhanced this immunofluorescence, whereas XAV939 reduced it. Interestingly, the reduction caused by AβOs alone was attenuated by LiCl and enhanced by XAV939 (Figure 7D).

### 3.7 Correlations between the α7 nAChR level and spatial learning and memory

Correlation analysis revealed a negative association between the elevated level of α7 nAChR protein present in the cerebral zone of APP/PS1 mice aged 6 (Figure S3A,C) or 12 (Figure S3B,D) months following exposure to LiCl and the impairment in their spatial learning and memory.

## 4 DISCUSSION

In the cerebral zone of APP/PS1 mice, one most common animal AD model, the content of Aβ and number of senile plaques are elevated, while the learning capability and memory function of these mice are impaired.^34^ Here, we found that in the cerebral zone of 6‐ and, especially, 12‐month‐old mice of this strain, the increases in size and quantity of senile plaques were evident and learning and memory significantly impaired. Consistent with previous studies,^25^, ^26^ we have also confirmed that Aβ damages neurons directly, both in vitro and in vivo, leading to dysfunction and apoptosis.

α7 nAChR is widespread throughout the central nervous system. Regarding the well‐defined intracerebral functions of α7 nAChR, it can modulate synaptic plasticity and transmission that underlay ordinary processes of cognition, attention, learning and memory.^35^, ^36^ In our current investigation, the protein expression of α7 nAChR in the cerebral hippocampus and cortex of APP/PS1 mice aged 6 and 12 months was found to be lower than in WT animals. In addition, primary neurons exposed to AβOs show a tendency towards reduced expression of α7 nAChR. Furthermore, we confirmed the damaging effect of Aβ on α7 nAChR both in vivo and in vitro, as well as the relationship between the loss of α7 nAChR and the decline in the learning capability and memory function for APP/PS1 mice. Computational modelling indicates that arginine‐208 and glutamate‐211 in α7 nAChR are involved in their interaction with Aβ.^37^ Moreover, our previous results showed that the loss of the ability of learning and memory of APP/PS1 mice can be reversed or aggravated via activation or suppression of α7 nAChR expression, respectively.^38^

At the same time, we found that the loss of α7 nAChR is associated with a reduction in the level of the β‐catenin protein. The decline of learning and memory in patients with AD also appears to be associated with the decrease of Wnt/β‐catenin in their brain.^39^ In animal models as well, the AD pathology is strongly correlated with the down‐regulation of Wnt/β‐catenin axis.^12^ Accordingly, the Wnt/β‐catenin signal pathway offers a therapeutic target for alleviating the pathological process that results in AD.^40^ In the present case, we verified that Wnt/β‐catenin signalling in the APP/PS1 murine cerebral zone or in the primary neurons exposed to AβOs *in vitro* is evidently weaker than in the WT mice or unexposed cells.

Evidence for a neuroprotective effect of lithium has accumulated over the last two decades,^41^ and this element has been found to be involved in the regulation of numerous genes, proteins and metabolites.^42^ Most noteworthy of these is the inhibition of GSK3β by lithium, which has a wide range of beneficial effects.^43^ Consistent with previous findings,^44^ we found here that LiCl enhances the learning capability and memory function of APP/PS1 mice aged both 6 and 12 months.

Moreover, both the elevated content of Aβ_42_ and the size or quantity of senile plaques in the cerebral zone of these transgenic mice were reduced through treatment with LiCl. In addition, this reduction in Aβ content was correlated to an improvement in learning and memory. Some evidence from animal models of AD indicates that LiCl can prevent neurotoxicity by reducing the production of Aβ,^45^ as well as that the cognitive benefits of LiCl are associated with enhanced clearance of Aβ via up‐regulation of brain microvascularization by lipoprotein receptor‐related protein 1 and increased bulk flow of cerebrospinal fluid.^46^ In human neuronal cells, LiCl alters proteins such as the rab proteins, which have been implicated in the processing of APP in connection with the pathophysiology of AD.^47^ In addition, up‐regulation of Wnt/β‐catenin signalling by LiCl can cause β‐catenin to bind specifically to regions within the BACE1 promoter that contain putative TCF/LEF motifs and repress transcription.^33^ Any of these mechanisms may explain the reduction in Aβ caused by lithium.

LiCl is a classic agonist of the Wnt/β‐catenin pathway, which is the main reason it has been chosen for testing as a candidate drug for treating AD in numerous studies.^10^, ^22^ Here, up‐regulation of the Wnt/β‐catenin axis by LiCl in 6‐ and 12‐month‐old mice with or without the APP/PS1 double mutation was verified. This effect was associated with inactivation of GSK3β through specific phosphorylation of Ser9 in its N‐terminus,^48^ which elevates the levels of the β‐catenin and cyclin D1 proteins. In addition, we found here that in primary hippocampal neurons the Wnt/β‐catenin pathway was activated by LiCl and inactivated by XAV939 or AβOs. Importantly, this effect by AβOs could be attenuated by LiCl, but aggravated by XAV939.

In line with these findings in vitro, the Wnt/β‐catenin axis initiation has been proven to offset the harmful action of AβO,^40^ which thus exerts a probable fundamental effect on maintaining neuronal and synaptic functions.^49^ Abundant evidence demonstrates that reactivation of the Wnt/β‐catenin pathway completely restores the number of synapses and synaptic plasticity^50^ and this pathway is a target for recovery of neuronal circuits following degeneration of synapses.^51^ Since α7 nAChRs are expressed widely on both pre‐synaptic and post‐synaptic membranes,^14^, ^15^ we hypothesize that activation of Wnt/β‐catenin pathway by LiCl stabilizes expression of these receptors in the animal AD models’ cerebral region.

Interestingly, the reduction in the cerebral α7 nAChR protein expression could be mitigated by LiCl exposure for the dual‐transgenic mice. In addition, in WT animals, treatment with LiCl led to elevated cortical or hippocampal levels of α7 nAChR protein and mRNA apart from initiating the Wnt/β‐catenin axis. These findings indicate that in the course of AD development, LiCl may regulate the α7 nAChR subunit expression. In this connection, negative association was noted between the increased protein level of α7 nAChR in the LiCl‐treated APP/PS1 murine brains and the impaired spatial learning and memory capabilities of these mice. In combination with previous observations, this study further supports the important role of α7 nAChR in learning and memory^35^, ^36^ and suggests that restoring normal expression of α7 nAChR may be one mechanism by which LiCl improves learning capability and memory function for APP/PS1 mice.

Subsequently, in primary neurons, it was found that the decrease in the α7 nAChR subunit expression in these cells induced by AβOs was attenuated by LiCl, but aggravated by XAV939. Furthermore, transfection of primary neurons with siRNA targeting the β‐catenin gene lowered not only the level of this protein and its downstream protein cyclin D1, but also reduced the α7 nAChR subunit levels. The above findings imply that the gene encoding α7 nAChR is regulated by the Wnt/β‐catenin pathway and that the reductions in α7 nAChR protein and mRNA induced by AβO may be mediated by inactivation of this pathway.

Cholinergic neurons are reliant on (α7) 5 nAChRs for maintaining the ability to learn and memory.^52^ At present, relatively little is known about potential interactions between the Wnt/β‐catenin pathway and the function of nAChRs. Earlier investigations revealed that the canonical Wnt axis initiation may promote the pre‐synaptic localization of α7‐nAChR^53^ and accelerate differentiation of stem cells in adipose tissue into cholinergic neurons.^54^ Moreover, activating the canonical Wnt pathway by Wnt‐7a has been reported to promote up‐regulation and aggregation of α7 nAChR.^55^ These observations are in line with the finding with *C*. *elegans* that mutations causing defects in the Wnt axis lower the synaptic expression of the ACR‐16/α7 nAChR homolog, a change accompanied by attenuated receptor function and loss of nicotinic‐related behaviors.^56^

Interestingly, a possible role for the expression and activity of N‐methyl‐d‐aspartic acid receptors (NMDARs) and glutamatergic transmission was found in the therapeutic effects of lithium.^57^ In many cases, the α7 nAChRs and NMDARs were closely apposed on axon terminals, where α7 nAChRs may regulate glutamate release and its subsequent modulation of neuronal plasticity.^58^, ^59^ Activating α7 nAChRs potentiated responses to glutamate by increasing the membrane insertion of NMDARs.^60^ However, whether lithium affects α7 nAChRs signal transduction through NMDARs has not been verified, which is worth of further study.

In the cerebral zone of APP/PS1 mice, the Aβ level was increased and those of α7 nAChR, phosphor‐GSK3β (ser9), β‐catenin and cyclin D1 (protein and/or mRNA levels) were reduced. Treatment with LiCl for 2 months at 4 or 10 months of age attenuated all of these effects. Similar expression variations were observed for these proteins in primary neurons exposed to AβOs, and these effects were attenuated by LiCl and aggravated by XAV939. Inhibition of β‐catenin expression lowered the level of α7 nAChR protein in these cells. A proposed molecular pathway involving above changes is shown in Figure S4. These findings suggest that LiCl can reverse the impairment in learning capability and memory function for APP/PS1 mice via a mechanism that might involve elevation of the level of α7 nAChR as a result of altered Wnt/β‐catenin signalling.

## CONFLICT OF INTEREST

The authors have no conflict of interest to declare.

## AUTHOR CONTRIBUTIONS

Jie Xiang: Formal analysis (equal); Writing‐original draft (equal). Longyan Ran: Formal analysis (equal). Xiaoxiao Zeng: Formal analysis (equal). Wenwen He: Formal analysis (equal). Yi Xu: Formal analysis (equal). Kun Cao: Formal analysis (equal). Yangting Dong: Formal analysis (equal). Xiaolan Qi: Formal analysis (equal). Wenfeng Yu: Formal analysis (equal). Yan Xiao: Formal analysis (equal). Zhizhong Guan: Conceptualization (equal); Writing‐review & editing (equal).

## Supporting information

Supplementary Material

## Data Availability

All data generated or analysed during this study are included in this published article and its supplementary information files.

## References

[1] Scheltens P , De Strooper B , Kivipelto M , et al. Alzheimer's disease. Lancet. 2021;397:1577‐1590. 33667416 10.1016/S0140-6736(20)32205-4 PMC8354300

[2] Lashley T , Schott JM , Weston P , et al. Molecular biomarkers of Alzheimer's disease: progress and prospects. Dis Model Mech. 2018;11:doi: 10.1242/dmm.031781 PMC5992610 29739861

[3] Hampel H , Mesulam M‐M , Cuello AC , et al. The cholinergic system in the pathophysiology and treatment of Alzheimer's disease. Brain. 2018;141:1917‐1933. 29850777 10.1093/brain/awy132 PMC6022632

[4] Mitsushima D , Sano A , Takahashi T . A cholinergic trigger drives learning‐induced plasticity at hippocampal synapses. Nat Commun. 2013;4:2760. 24217681 10.1038/ncomms3760 PMC3831287

[5] Lombardo S , Maskos U . Role of the nicotinic acetylcholine receptor in Alzheimer's disease pathology and treatment. Neuropharmacology. 2015;96:255‐262. 25514383 10.1016/j.neuropharm.2014.11.018

[6] Guan Z‐Z , Zhang X , Ravid R , et al. Decreased protein levels of nicotinic receptor subunits in the hippocampus and temporal cortex of patients with Alzheimer's disease. J Neurochem. 2000;74:237‐243. 10617125 10.1046/j.1471-4159.2000.0740237.x

[7] Guan ZZ , Miao H , Tian JY , et al. Suppressed expression of nicotinic acetylcholine receptors by nanomolar beta‐amyloid peptides in PC12 cells. J Neural Transm (Vienna). 2001;108:1417‐1433. 11810405 10.1007/s007020100017

[8] Pym L , Kemp M , Raymond‐Delpech V , et al. Subtype‐specific actions of beta‐amyloid peptides on recombinant human neuronal nicotinic acetylcholine receptors (alpha7, alpha4beta2, alpha3beta4) expressed in *Xenopus laevis* oocytes. Br J Pharmacol. 2005;146:964‐971. 16184187 10.1038/sj.bjp.0706403 PMC1751230

[9] Wang HY , Lee DH , D'Andrea MR , et al. beta‐Amyloid(1–42) binds to alpha7 nicotinic acetylcholine receptor with high affinity. Implications for Alzheimer's disease pathology. J Biol Chem. 2000;275:5626‐5632. 10681545 10.1074/jbc.275.8.5626

[10] Clevers H , Nusse R . Wnt/β‐catenin signaling and disease. Cell. 2012;149:1192‐1205. 22682243 10.1016/j.cell.2012.05.012

[11] Menet R , Bourassa P , Calon F , et al. Dickkopf‐related protein‐1 inhibition attenuates amyloid‐beta pathology associated to Alzheimer's disease. Neurochem Int. 2020;141:104881. 33068684 10.1016/j.neuint.2020.104881

[12] Tapia‐Rojas C , Inestrosa NC . Loss of canonical Wnt signaling is involved in the pathogenesis of Alzheimer's disease. Neural Regen Res. 2018;13:1705‐1710. 30136680 10.4103/1673-5374.238606 PMC6128062

[13] Jia L , Piña‐Crespo J , Li Y . Restoring Wnt/β‐catenin signaling is a promising therapeutic strategy for Alzheimer's disease. Mol Brain. 2019;12:104. 31801553 10.1186/s13041-019-0525-5 PMC6894260

[14] Chu ZG , Zhou FM , Hablitz JJ . Nicotinic acetylcholine receptor‐mediated synaptic potentials in rat neocortex. Brain Res. 2000;887:399‐405. 11134630 10.1016/s0006-8993(00)03076-6

[15] Zolles G , Wagner E , Lampert A , et al. Functional expression of nicotinic acetylcholine receptors in rat neocortical layer 5 pyramidal cells. Cereb Cortex. 2009;19:1079‐1091. 18794204 10.1093/cercor/bhn158

[16] Vallée A , Vallée J‐N , Guillevin R , et al. Riluzole: a therapeutic strategy in Alzheimer's disease by targeting the WNT/β‐catenin pathway. Aging (Albany NY). 2020;12:3095‐3113. 32035419 10.18632/aging.102830 PMC7041777

[17] Jin NA , Zhu H , Liang X , et al. Sodium selenate activated Wnt/β‐catenin signaling and repressed amyloid‐β formation in a triple transgenic mouse model of Alzheimer's disease. Exp Neurol. 2017;297:36‐49. 28711506 10.1016/j.expneurol.2017.07.006

[18] Nunes PV , Forlenza OV , Gattaz WF . Lithium and risk for Alzheimer's disease in elderly patients with bipolar disorder. Br J Psychiatry. 2007;190:359‐360. 17401045 10.1192/bjp.bp.106.029868

[19] Rybakowski JK . Challenging the negative perception of lithium and optimizing its long‐term administration. Front Mol Neurosci. 2018;11:349. 30333722 10.3389/fnmol.2018.00349 PMC6175994

[20] Wen J , Sawmiller D , Wheeldon B , et al. A review for Lithium: pharmacokinetics, drug design, and toxicity. CNS Neurol Disord Drug Targets. 2019;18:769‐778. 31724518 10.2174/1871527318666191114095249

[21] Hampel H , Lista S , Mango D , et al. Lithium as a treatment for Alzheimer's disease: the systems pharmacology perspective. J Alzheimers Dis. 2019;69:615‐629. 31156173 10.3233/JAD-190197

[22] Cisternas P , Zolezzi JM , Martinez M , et al. Wnt‐induced activation of glucose metabolism mediates the in vivo neuroprotective roles of Wnt signaling in Alzheimer disease. J Neurochem. 2019;149:54‐72. 30300917 10.1111/jnc.14608 PMC7680578

[23] Shetti D , Zhang B , Fan C , et al. Low dose of paclitaxel combined with XAV939 attenuates metastasis, angiogenesis and growth in breast cancer by suppressing Wnt signaling. Cells. 2019;8:892. 10.3390/cells8080892 PMC6721645 31416135

[24] Zhao L , Gong N , Liu M , et al. Beneficial synergistic effects of microdose lithium with pyrroloquinoline quinone in an Alzheimer's disease mouse model. Neurobiol Aging. 2014;35:2736‐2745. 25018109 10.1016/j.neurobiolaging.2014.06.003

[25] Xiang J , Cao K , Dong Y‐T , et al. Lithium chloride reduced the level of oxidative stress in brains and serums of APP/PS1 double transgenic mice via the regulation of GSK3β/Nrf2/HO‐1 pathway. Int J Neurosci. 2020;130:564‐573. 31679397 10.1080/00207454.2019.1688808

[26] Dong Y‐T , Cao K , Tan L‐C , et al. Stimulation of SIRT1 attenuates the level of oxidative stress in the brains of APP/PS1 double transgenic mice and in primary neurons exposed to oligomers of the amyloid‐β peptide. J Alzheimers Dis. 2018;63:283‐301. 29614660 10.3233/JAD-171020

[27] Klein WL . Abeta toxicity in Alzheimer's disease: globular oligomers (ADDLs) as new vaccine and drug targets. Neurochem Int. 2002;41:345‐352. 12176077 10.1016/s0197-0186(02)00050-5

[28] Berardo C , Siciliano V , Di Pasqua LG , et al. Comparison between lipofectamine RNAiMAX and GenMute transfection agents in two cellular models of human hepatoma. Eur J Histochem. 2019;63: doi: 10.4081/ejh.2019.3048 PMC6712361 31455073

[29] Cao K , Xiang J , Dong Y‐T , et al. Exposure to fluoride aggravates the impairment in learning and memory and neuropathological lesions in mice carrying the APP/PS1 double‐transgenic mutation. Alzheimers Res Ther. 2019;11:35. 31010414 10.1186/s13195-019-0490-3 PMC6477877

[30] Duan JJ , Zhang Q , Hu X , et al. N(4)‐acetylcytidine is required for sustained NLRP3 inflammasome activation via HMGB1 pathway in microglia. Cell Signal. 2019;58:44‐52. 30853521 10.1016/j.cellsig.2019.03.007

[31] Cao K , Dong Y‐T , Xiang J , et al. Reduced expression of SIRT1 and SOD‐1 and the correlation between these levels in various regions of the brains of patients with Alzheimer's disease. J Clin Pathol. 2018;71:1090‐1099. 30185534 10.1136/jclinpath-2018-205320

[32] Yamamoto T , Hirano A . A comparative study of modified Bielschowsky, Bodian and thioflavin S stains on Alzheimer's neurofibrillary tangles. Neuropathol Appl Neurobiol. 1986;12:3‐9. 2422580 10.1111/j.1365-2990.1986.tb00677.x

[33] Parr C , Mirzaei N , Christian M , et al. Activation of the Wnt/β‐catenin pathway represses the transcription of the β‐amyloid precursor protein cleaving enzyme (BACE1) via binding of T‐cell factor‐4 to BACE1 promoter. FASEB J. 2015;29:623‐635. 25384422 10.1096/fj.14-253211

[34] Shi Q , Chowdhury S , Ma R , et al. Complement C3 deficiency protects against neurodegeneration in aged plaque‐rich APP/PS1 mice. Sci Transl Med. 2017;9:doi: 10.1126/scitranslmed.aaf6295 PMC6936623 28566429

[35] Kabbani N , Nichols RA . Beyond the channel: metabotropic signaling by nicotinic receptors. Trends Pharmacol Sci. 2018;39:354‐366. 29428175 10.1016/j.tips.2018.01.002

[36] Martín‐Sánchez C , Alés E , Balseiro‐Gómez S , et al. The human‐specific duplicated α7 gene inhibits the ancestral α7, negatively regulating nicotinic acetylcholine receptor‐mediated transmitter release. J Biol Chem. 2021;296:100341. 33515545 10.1016/j.jbc.2021.100341 PMC7949125

[37] Roberts JP , Stokoe SA , Sathler MF , et al. Selective coactivation of α7‐ and α4β2‐nicotinic acetylcholine receptors reverses beta‐amyloid‐induced synaptic dysfunction. J Biol Chem. 2021;296:100402. 33571523 10.1016/j.jbc.2021.100402 PMC7961090

[38] Cao K , Dong Y‐T , Xiang J , et al. The neuroprotective effects of SIRT1 in mice carrying the APP/PS1 double‐transgenic mutation and in SH‐SY5Y cells over‐expressing human APP670/671 may involve elevated levels of α7 nicotinic acetylcholine receptors. Aging (Albany NY). 2020;12:1792‐1807. 32003755 10.18632/aging.102713 PMC7053601

[39] Folke J , Pakkenberg B , Brudek T . Impaired Wnt signaling in the prefrontal cortex of Alzheimer's disease. Mol Neurobiol. 2019;56:873‐891. 29804228 10.1007/s12035-018-1103-z

[40] Huang M , Liang Y , Chen H , et al. The role of fluoxetine in activating Wnt/β‐catenin signaling and repressing β‐amyloid production in an Alzheimer mouse model. Front Aging Neurosci. 2018;10:164. 29910725 10.3389/fnagi.2018.00164 PMC5992518

[41] Rybakowski JK , Suwalska A , Hajek T . Clinical perspectives of Lithium's neuroprotective effect. Pharmacopsychiatry. 2018;51:194‐199. 29270949 10.1055/s-0043-124436

[42] Roux M , Dosseto A . From direct to indirect lithium targets: a comprehensive review of omics data. Metallomics. 2017;9:1326‐1351. 28885630 10.1039/c7mt00203c

[43] Sofola‐Adesakin O , Castillo‐Quan JI , Rallis C , et al. Lithium suppresses Aβ pathology by inhibiting translation in an adult Drosophila model of Alzheimer's disease. Front Aging Neurosci. 2014;6:190. 25126078 10.3389/fnagi.2014.00190 PMC4115666

[44] Rivera DS , Lindsay C , Codocedo JF , et al. Andrographolide recovers cognitive impairment in a natural model of Alzheimer's disease (*Octodon degus*). Neurobiol Aging. 2016;46:204‐220. 27505720 10.1016/j.neurobiolaging.2016.06.021

[45] Phiel CJ , Wilson CA , Lee VM , et al. GSK‐3alpha regulates production of Alzheimer's disease amyloid‐beta peptides. Nature. 2003;423:435‐439. 12761548 10.1038/nature01640

[46] Pan Y , Short JL , Newman SA , et al. Cognitive benefits of lithium chloride in APP/PS1 mice are associated with enhanced brain clearance of β‐amyloid. Brain Behav Immun. 2018;70:36‐47. 29545118 10.1016/j.bbi.2018.03.007

[47] Thompson AJ , Williamson R , Schofield E , et al. Quantitation of glycogen synthase kinase‐3 sensitive proteins in neuronal membrane rafts. Proteomics. 2009;9:3022‐3035. 19526546 10.1002/pmic.200900006

[48] Sutherland C , Leighton IA , Cohen P . Inactivation of glycogen synthase kinase‐3 beta by phosphorylation: new kinase connections in insulin and growth‐factor signalling. Biochem J. 1993;296(Pt 1):15‐19. 8250835 10.1042/bj2960015 PMC1137648

[49] Arrázola MS , Silva‐Alvarez C , Inestrosa NC . How the Wnt signaling pathway protects from neurodegeneration: the mitochondrial scenario. Front Cell Neurosci. 2015;9:166. 25999816 10.3389/fncel.2015.00166 PMC4419851

[50] Marzo A , Galli S , Lopes D , et al. Reversal of synapse degeneration by restoring Wnt signaling in the adult hippocampus. Curr Biol. 2016;26:2551‐2561. 27593374 10.1016/j.cub.2016.07.024 PMC5070786

[51] Cisternas P , Oliva CA , Torres VI , et al. Presymptomatic treatment with andrographolide improves brain metabolic markers and cognitive behavior in a model of early‐onset Alzheimer's disease. Front Cell Neurosci. 2019;13:295. 31379502 10.3389/fncel.2019.00295 PMC6657419

[52] Dineley KT . Beta‐amyloid peptide–nicotinic acetylcholine receptor interaction: the two faces of health and disease. Front Biosci. 2007;12:5030‐5038. 17569627 10.2741/2445

[53] Farías GG , Vallés AS , Colombres M , et al. Wnt‐7a induces presynaptic colocalization of alpha 7‐nicotinic acetylcholine receptors and adenomatous polyposis coli in hippocampal neurons. J Neurosci. 2007;27:5313‐5325. 17507554 10.1523/JNEUROSCI.3934-06.2007 PMC6672358

[54] Liu B , Deng C , Zhang Y , et al. Wnt3a expression during the differentiation of adipose‐derived stem cells into cholinergic neurons. Neural Regen Res. 2012;7:1463‐1468. 25657680 10.3969/j.issn.1673-5374.2012.19.003 PMC4308776

[55] Inestrosa NC , Godoy JA , Vargas JY , et al. Nicotine prevents synaptic impairment induced by amyloid‐β oligomers through α7‐nicotinic acetylcholine receptor activation. Neuromolecular Med. 2013;15:549‐569. 23842742 10.1007/s12017-013-8242-1

[56] Jensen M , Hoerndli FJ , Brockie P , et al. Wnt signaling regulates acetylcholine receptor translocation and synaptic plasticity in the adult nervous system. Cell. 2012;149:173‐187. 22464329 10.1016/j.cell.2011.12.038 PMC3375111

[57] Ghasemi M , Dehpour AR . The NMDA receptor/nitric oxide pathway: a target for the therapeutic and toxic effects of lithium. Trends Pharmacol Sci. 2011;32:420‐434. 21492946 10.1016/j.tips.2011.03.006

[58] Zappettini S , Grilli M , Olivero G , et al. Nicotinic α7 receptor activation selectively potentiates the function of NMDA receptors in glutamatergic terminals of the nucleus accumbens. Front Cell Neurosci. 2014;8:332. 25360085 10.3389/fncel.2014.00332 PMC4199379

[59] Salamone A , Zappettini S , Grilli M , et al. Prolonged nicotine exposure down‐regulates presynaptic NMDA receptors in dopaminergic terminals of the rat nucleus accumbens. Neuropharmacology. 2014;79:488‐497. 24373903 10.1016/j.neuropharm.2013.12.014

[60] Grilli M , Summa M , Salamone A , et al. In vitro exposure to nicotine induces endocytosis of presynaptic AMPA receptors modulating dopamine release in rat nucleus accumbens nerve terminals. Neuropharmacology. 2012;63:916‐926. 22771975 10.1016/j.neuropharm.2012.06.049

