# Supplementary figures and images for: LiCl attenuates impaired learning and memory of APP/PS1 mice, which in mechanism involves α7 nAChRs and Wnt/β‐catenin pathway
Source: J Cell Mol Med. 2021 Oct 28;25(22):10698–710. doi: 10.1111/jcmm.17006 (PMC8581309; doi:10.1111/jcmm.17006)

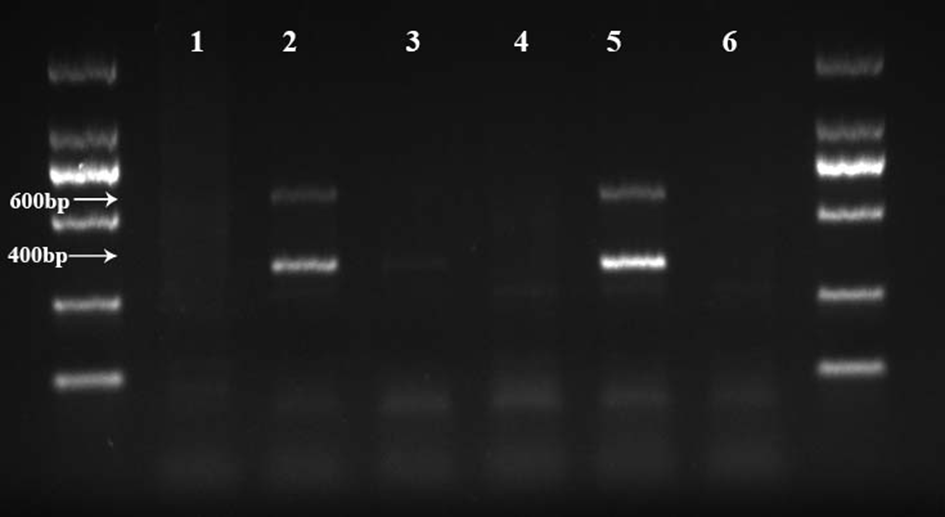

Supplement: Supplementary file 1 — Supplementary Material [file JCMM-25-10698-s001.zip › jcmm17006-sup-0001-FigS1.tif]

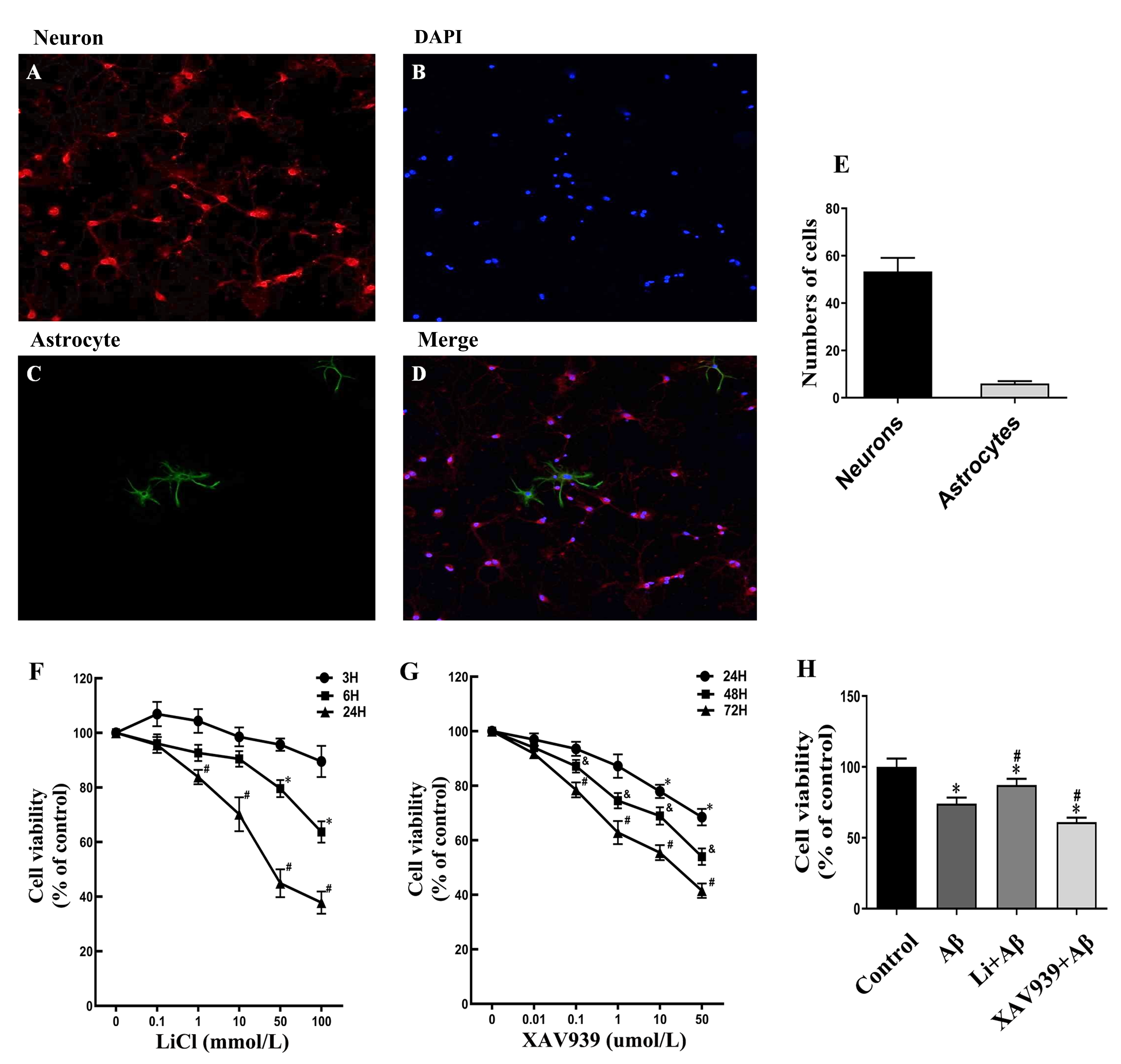

Supplement: Supplementary file 1 — Supplementary Material [file JCMM-25-10698-s001.zip › jcmm17006-sup-0002-FigS2.tif]

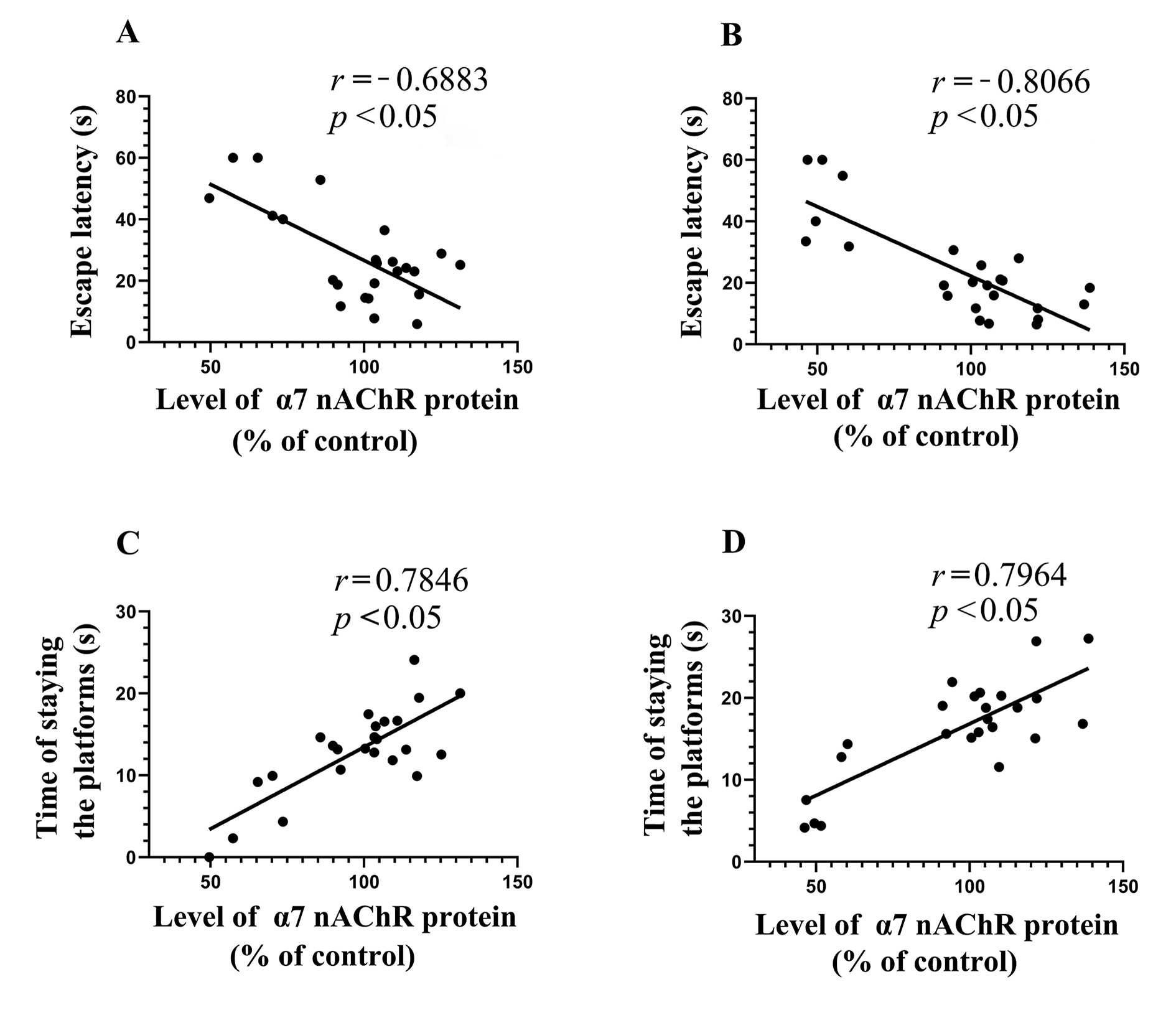

Supplement: Supplementary file 1 — Supplementary Material [file JCMM-25-10698-s001.zip › jcmm17006-sup-0003-FigS3.tif]

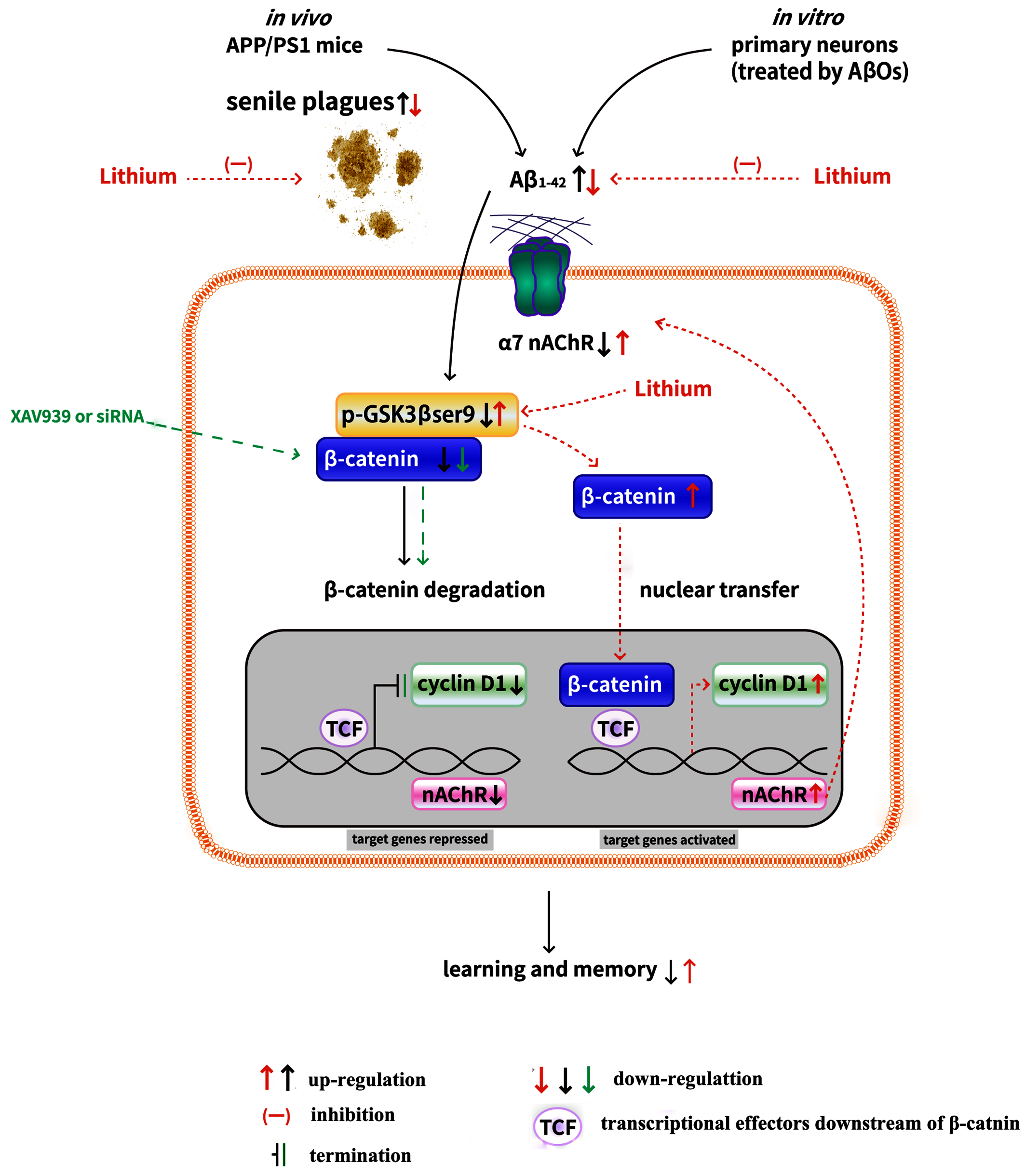

Supplement: Supplementary file 1 — Supplementary Material [file JCMM-25-10698-s001.zip › jcmm17006-sup-0004-FigS4.tif]

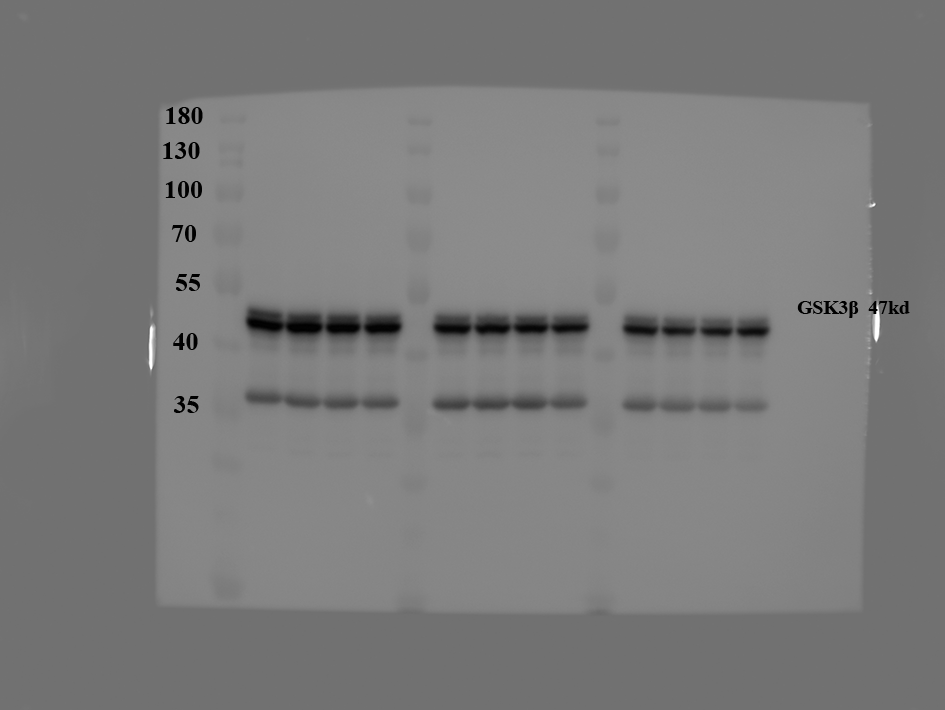

Supplement: Supplementary file 1 — Supplementary Material [file JCMM-25-10698-s001.zip › jcmm17006-sup-0005-FigS3d-1(raw data).tif]

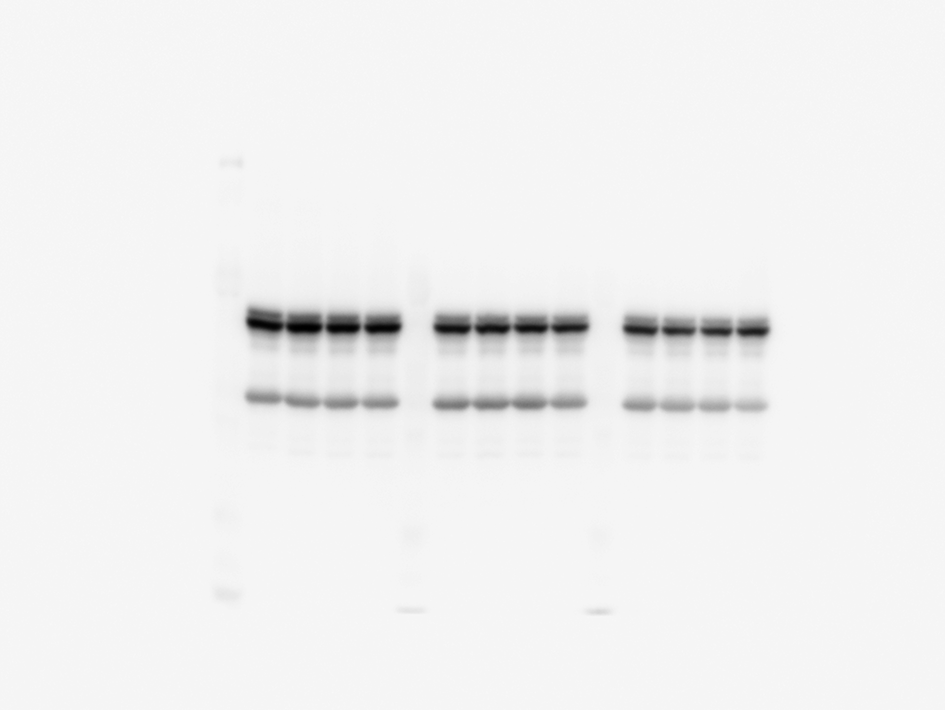

Supplement: Supplementary file 1 — Supplementary Material [file JCMM-25-10698-s001.zip › jcmm17006-sup-0006-FigS3d-2(raw data).tif]

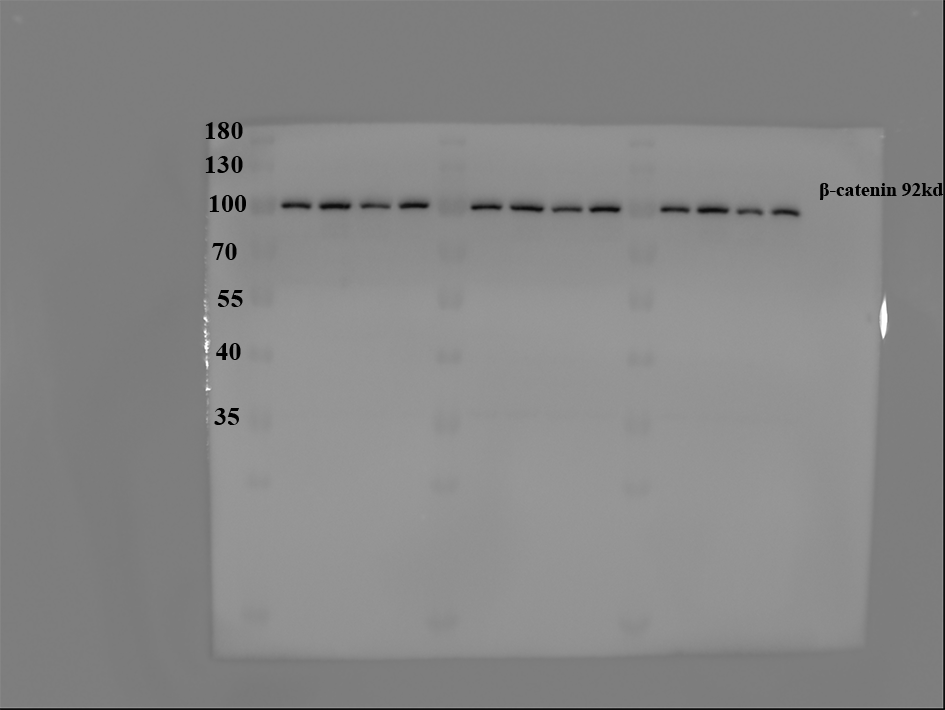

Supplement: Supplementary file 1 — Supplementary Material [file JCMM-25-10698-s001.zip › jcmm17006-sup-0007-FigS3e-1(raw data).tif]

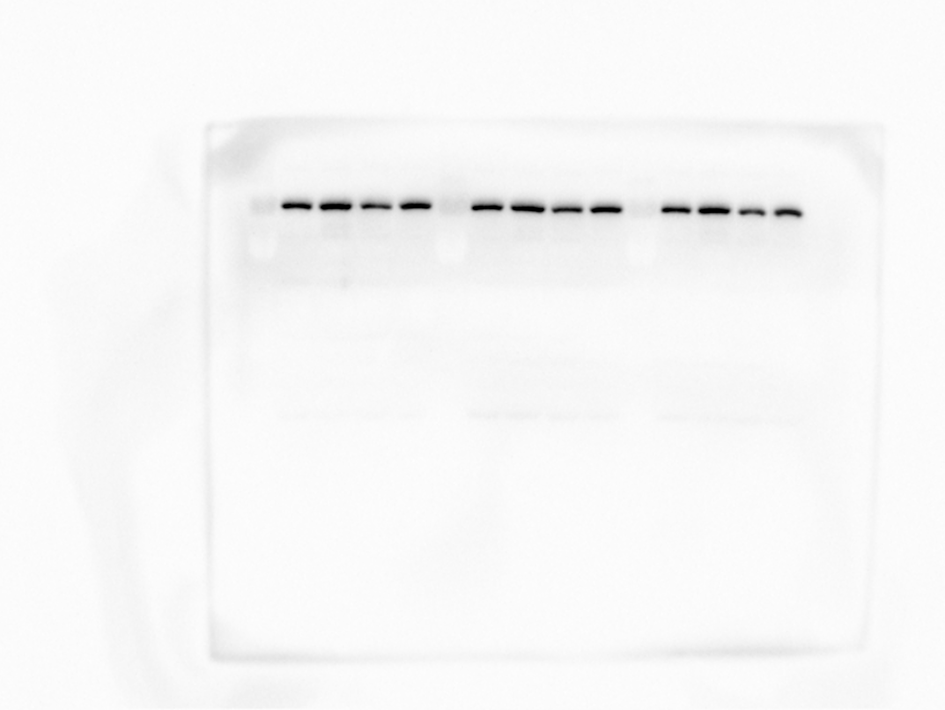

Supplement: Supplementary file 1 — Supplementary Material [file JCMM-25-10698-s001.zip › jcmm17006-sup-0008-FigS3e-2(raw data).tif]

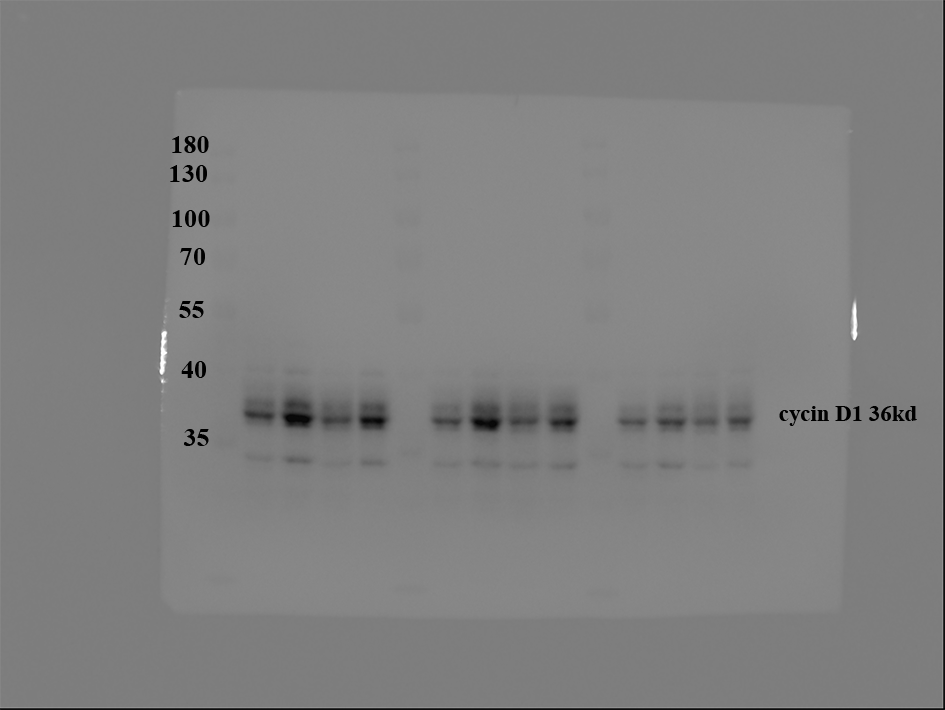

Supplement: Supplementary file 1 — Supplementary Material [file JCMM-25-10698-s001.zip › jcmm17006-sup-0009-Fig3f.-1(raw data).tif]

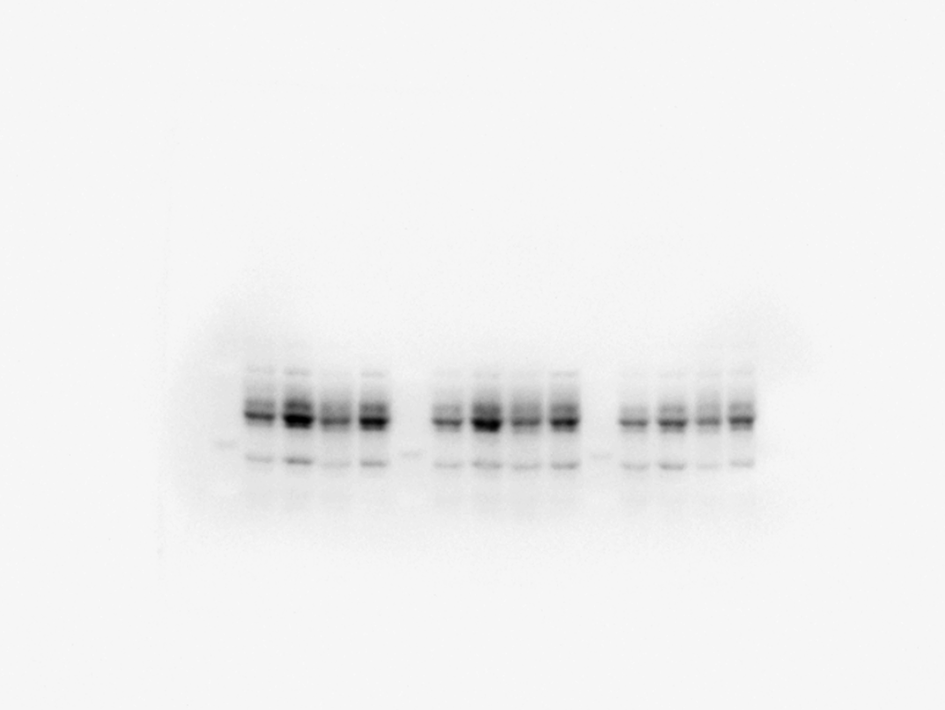

Supplement: Supplementary file 1 — Supplementary Material [file JCMM-25-10698-s001.zip › jcmm17006-sup-0010-Fig3f.-2(raw data).tif]

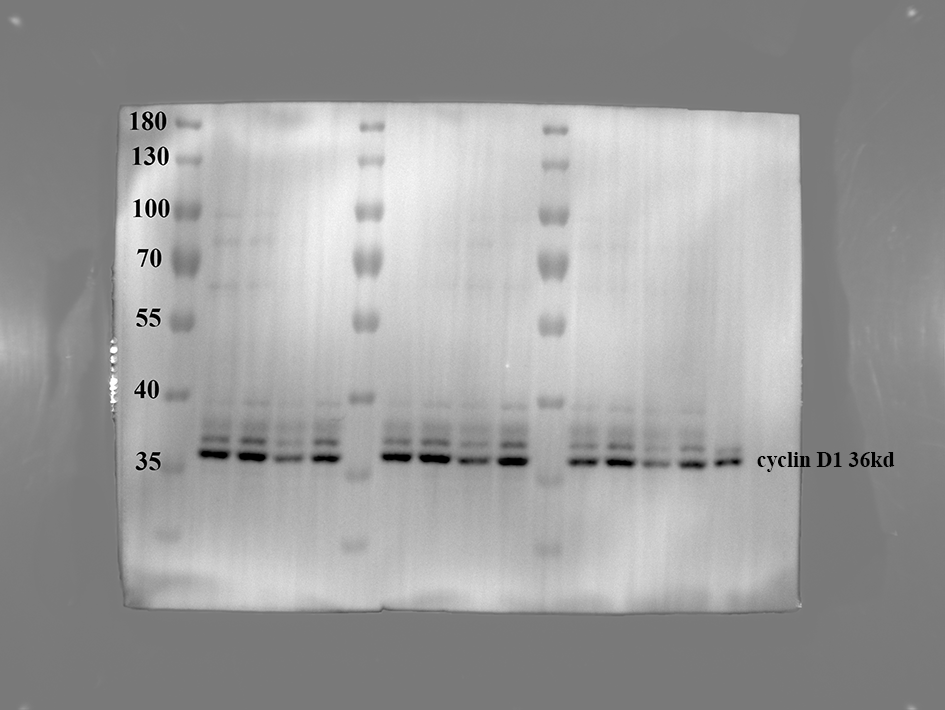

Supplement: Supplementary file 1 — Supplementary Material [file JCMM-25-10698-s001.zip › jcmm17006-sup-0011-Fig3F-1(raw data).tif]

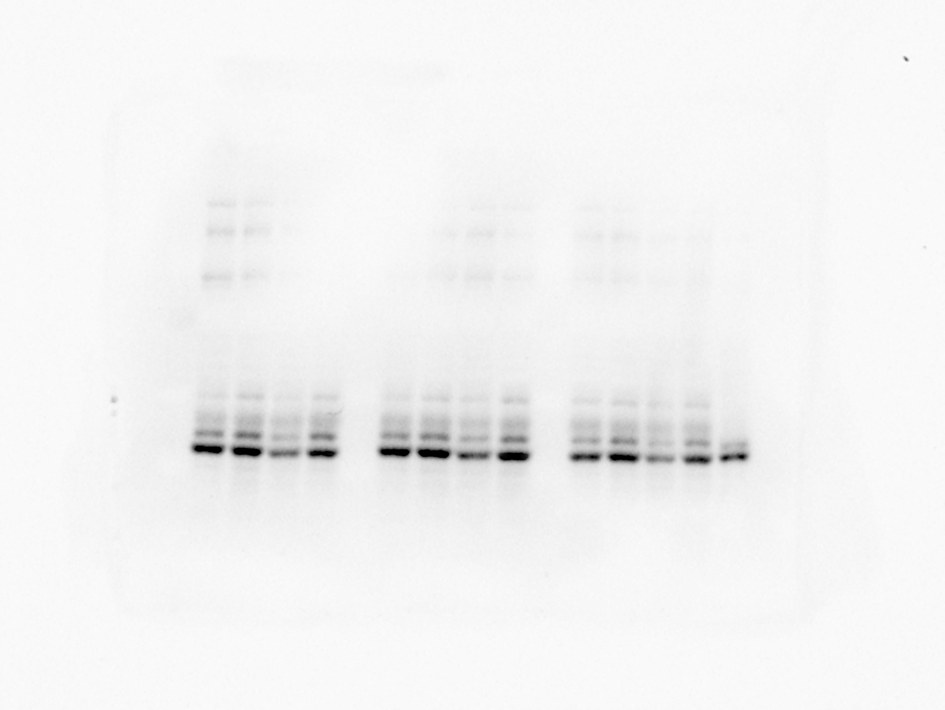

Supplement: Supplementary file 1 — Supplementary Material [file JCMM-25-10698-s001.zip › jcmm17006-sup-0012-Fig3F-2(raw data).tif]

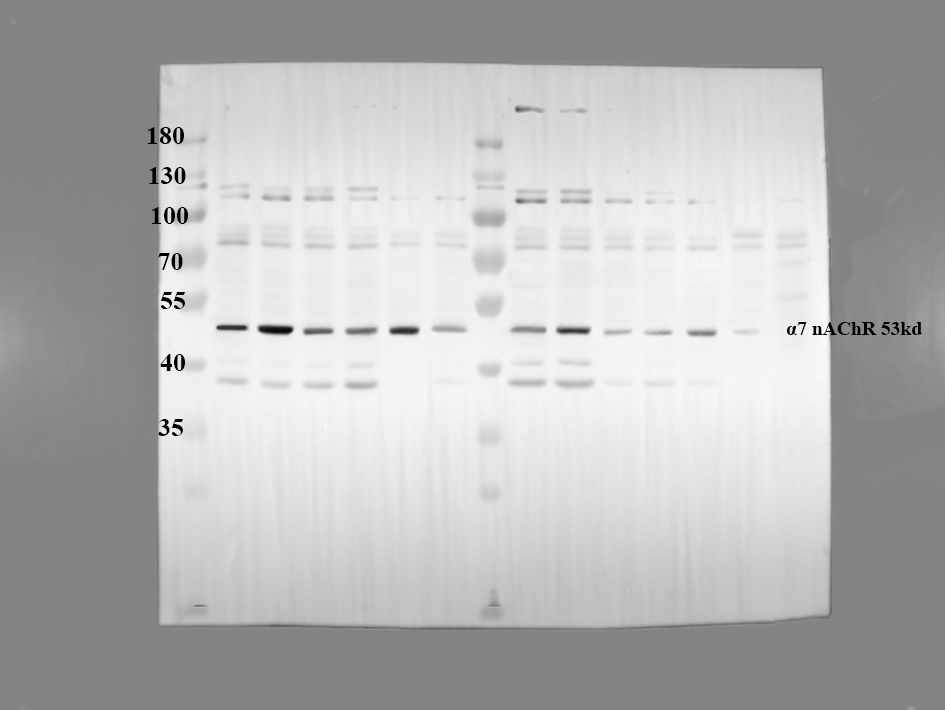

Supplement: Supplementary file 1 — Supplementary Material [file JCMM-25-10698-s001.zip › jcmm17006-sup-0013-Fig4C-1(raw data).tif]

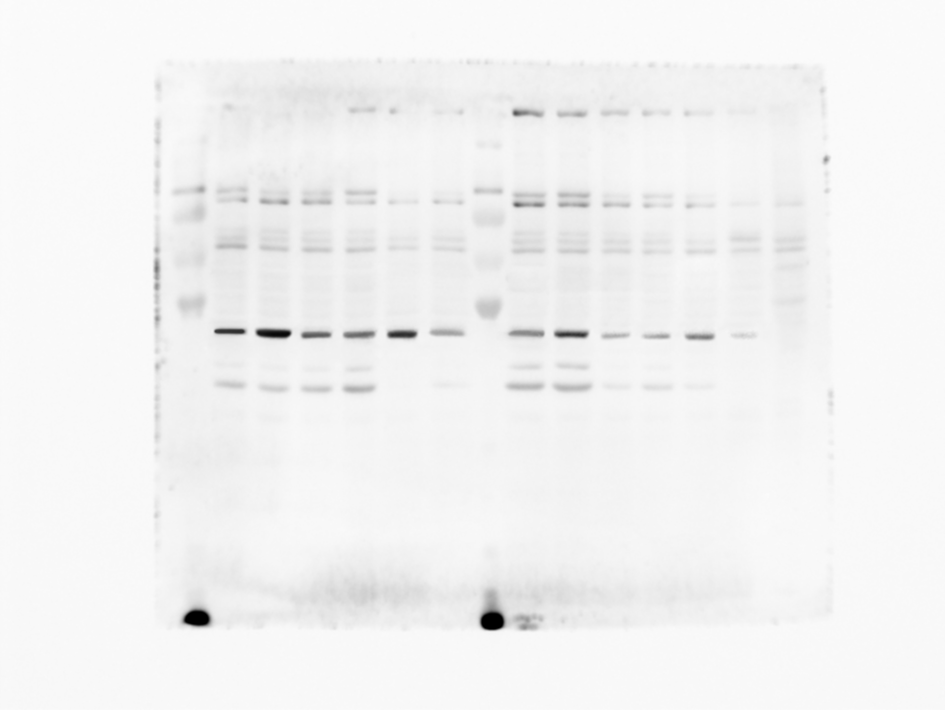

Supplement: Supplementary file 1 — Supplementary Material [file JCMM-25-10698-s001.zip › jcmm17006-sup-0014-Fig4C-2(raw data).tif]

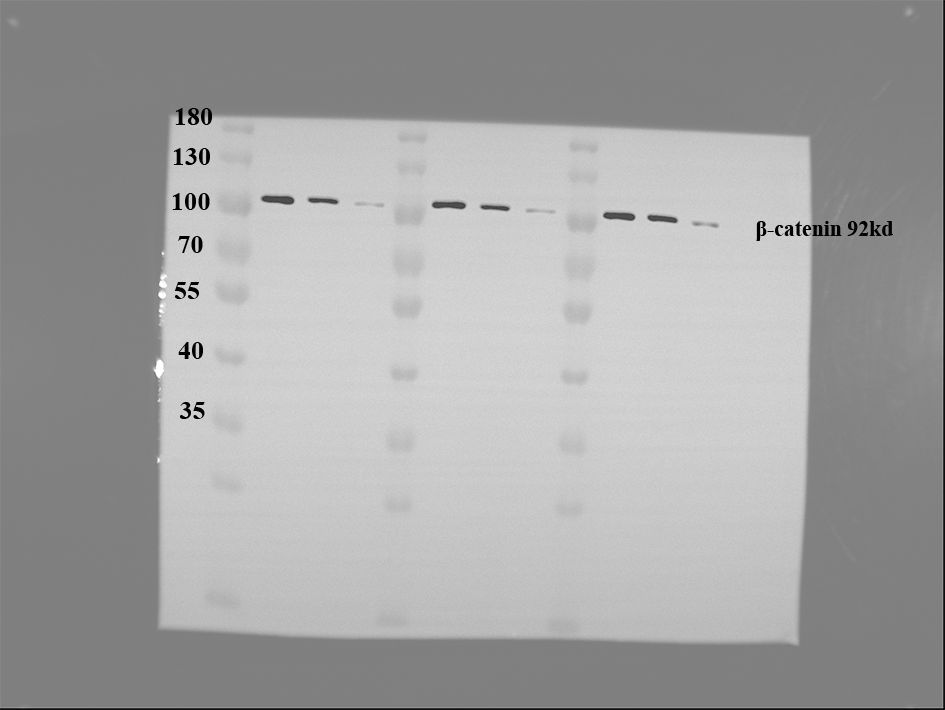

Supplement: Supplementary file 1 — Supplementary Material [file JCMM-25-10698-s001.zip › jcmm17006-sup-0015-Fig4E-1(raw data).tif]

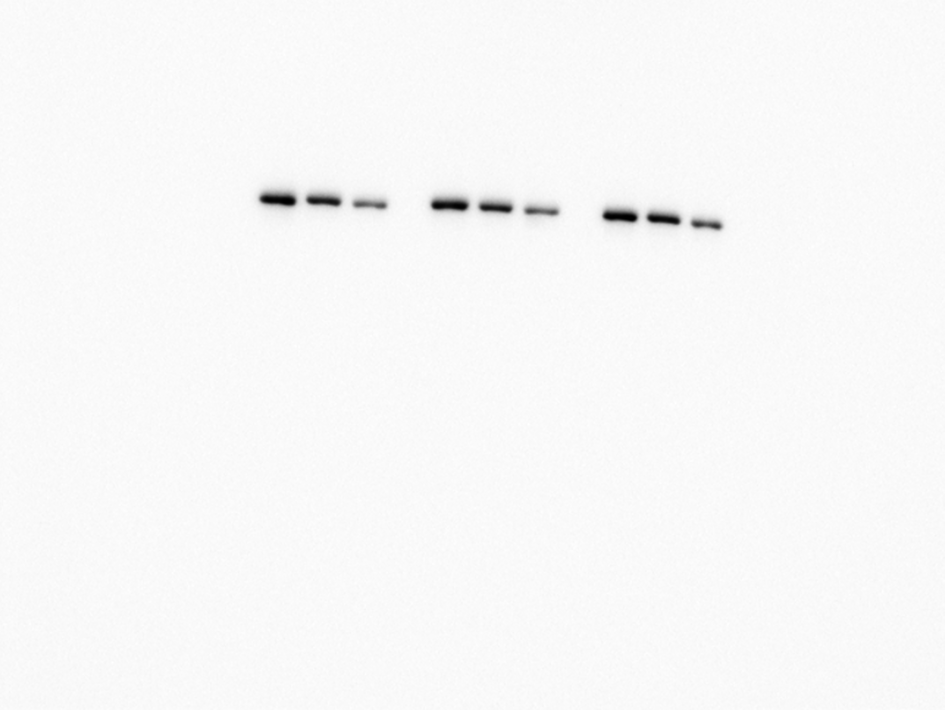

Supplement: Supplementary file 1 — Supplementary Material [file JCMM-25-10698-s001.zip › jcmm17006-sup-0016-Fig4E-2(raw data).tif]

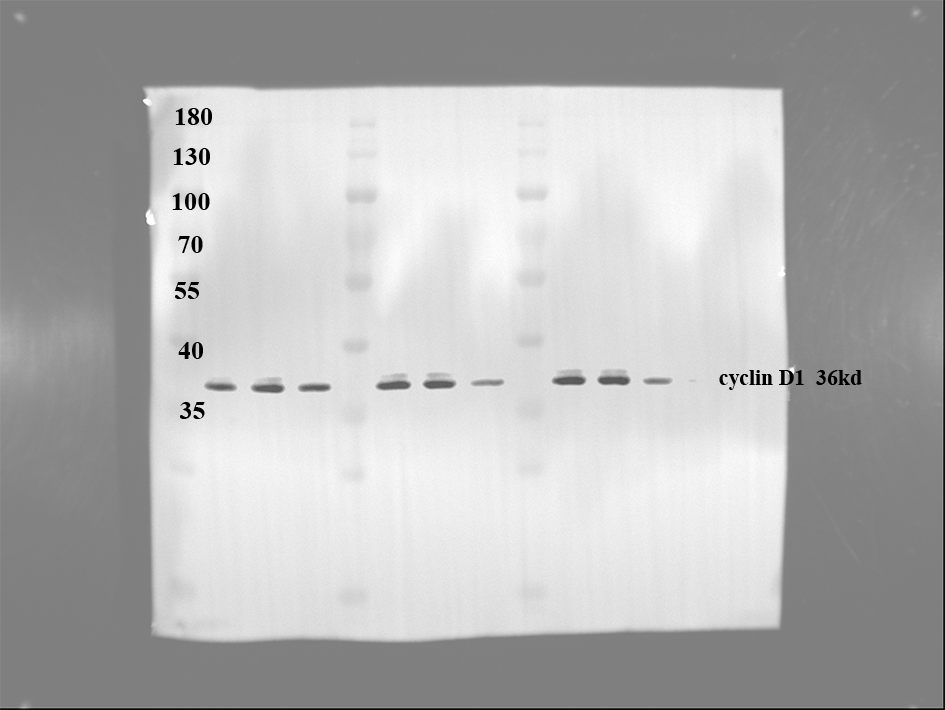

Supplement: Supplementary file 1 — Supplementary Material [file JCMM-25-10698-s001.zip › jcmm17006-sup-0017-Fig4F-1(raw data).tif]

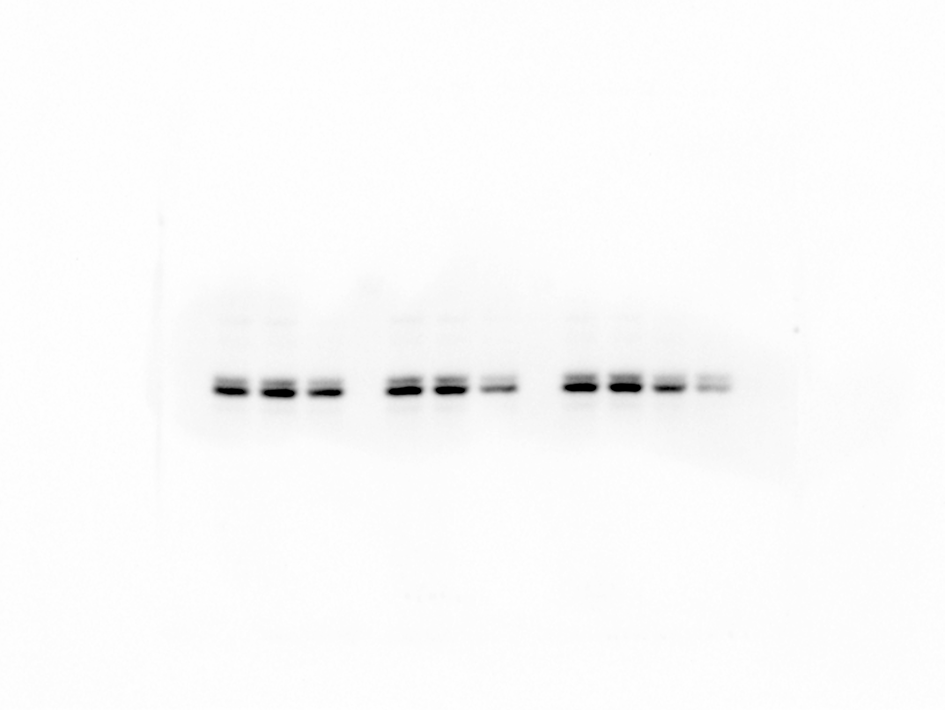

Supplement: Supplementary file 1 — Supplementary Material [file JCMM-25-10698-s001.zip › jcmm17006-sup-0018-Fig4F-2(raw data).tif]

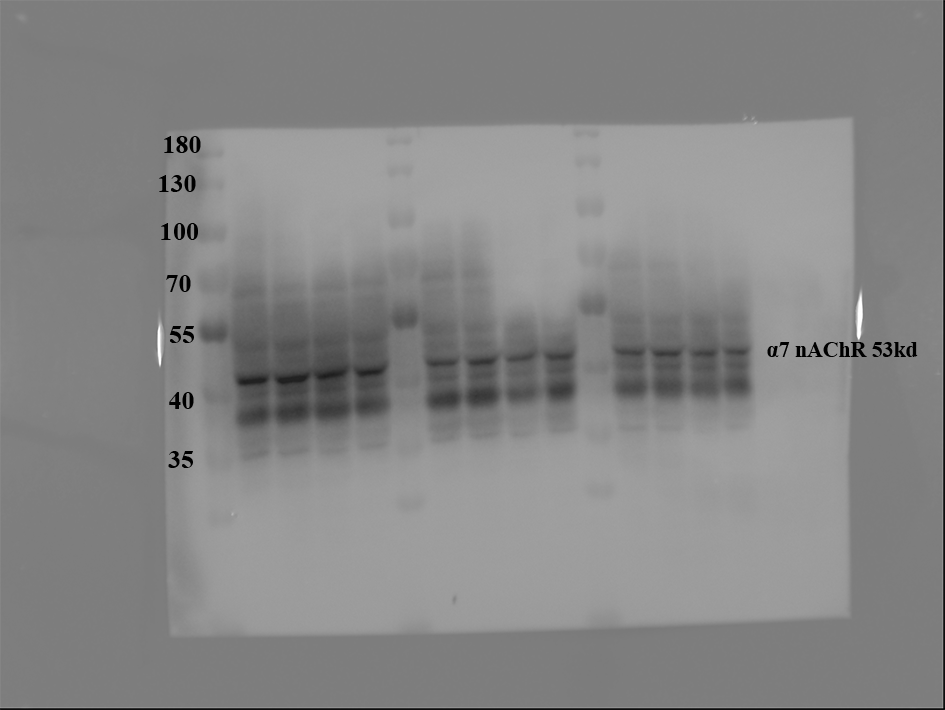

Supplement: Supplementary file 1 — Supplementary Material [file JCMM-25-10698-s001.zip › jcmm17006-sup-0019-Fig5D-1(raw data).tif]

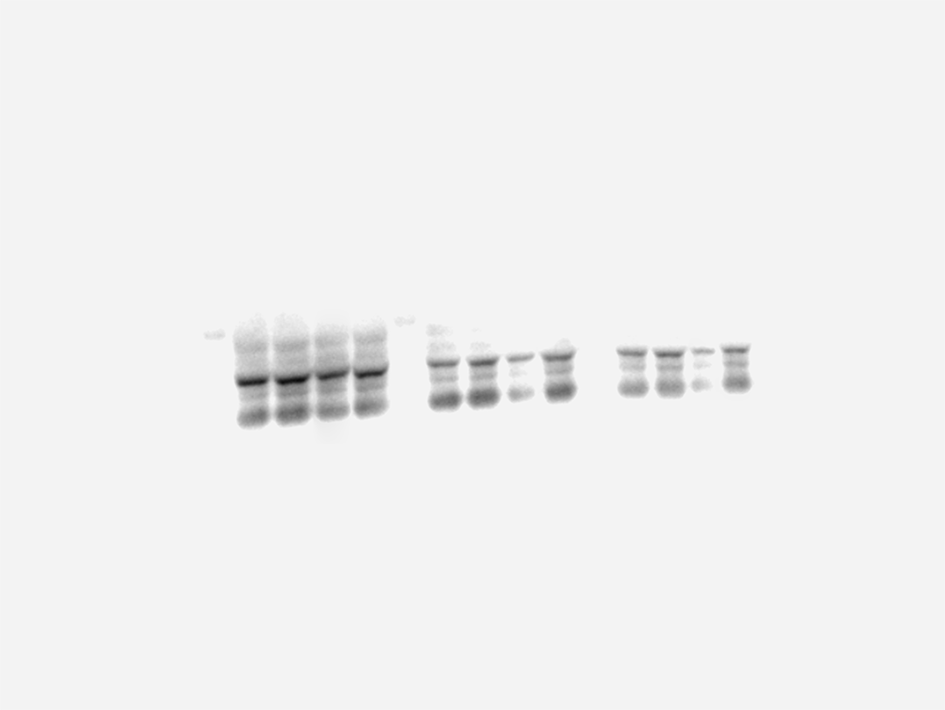

Supplement: Supplementary file 1 — Supplementary Material [file JCMM-25-10698-s001.zip › jcmm17006-sup-0020-Fig5D-2(raw data).tif]
